# ZNF582 overexpression restrains the progression of clear cell renal cell carcinoma by enhancing the binding of TJP2 and ERK2 and inhibiting ERK2 phosphorylation

**DOI:** 10.1038/s41419-023-05750-y

**Published:** 2023-03-25

**Authors:** Wuping Yang, Zedan Zhang, Lei Li, Kenan Zhang, Yawei Xu, Mancheng Xia, Jingcheng Zhou, Yanqing Gong, Jinchao Chen, Kan Gong

**Affiliations:** 1 Department of Urology, Peking University First Hospital, Beijing, 100034 P.R. China; 2 Hereditary Kidney Cancer Research Center, Peking University First Hospital, Beijing, 100034 P.R. China; 3 Beijing Key Laboratory of Urogenital Diseases (Male) Molecular Diagnosis and Treatment Center, Beijing, P.R. China; 4 Department of Urologic Surgery, The Cancer Hospital of the University of Chinese Academy of Sciences, Zhejiang Cancer Hospital, Institute of Basic Medicine and Cancer (IBMC), Chinese Academy of Sciences, Hangzhou, 310022 P.R. China

**Keywords:** Renal cell carcinoma, Tumour-suppressor proteins

## Abstract

Recent evidences have suggested that Zinc finger protein 582 (ZNF582) plays different important roles in various tumors, but its clinical role, biological function and regulatory mechanism in clear cell renal cell carcinoma (ccRCC) are still vague. Through analyzing GEO and TCGA-KIRC data and validation with local samples, we identified the low expression pattern of ZNF582 in ccRCC. Decreased ZNF582 expression is correlated with higher tumor stage and grade, distant metastasis and poor prognosis. By analyzing the DNA methylation data of ccRCC in TCGA-KIRC and using Massarray DNA methylation and demethylation analysis, we confirmed the hypermethylation status of ZNF582 in ccRCC and its negative regulation on ZNF582 expression. Using cell phenotype experiments and orthotopic kidney tumor growth models, we determined the inhibitory effect of ZNF582 overexpression on ccRCC growth and metastasis in vivo and in vitro. Mechanistically, using TMT (Tandem mass tags) quantitative proteomics test, Co-IP (Co-immunoprecipitation) and Western Blot experiments, we clarified that ZNF582 binds to TJP2 and up-regulates TJP2 protein expression. Increased TJP2 protein combines with ERK2 to promote ERK2 protein expression and suppresses the phosphorylation of ERK2, thereby inhibiting the growth and metastasis of ccRCC. In general, our findings provide the first solid theoretical rationale for targeting ZNF582/TJP2/ERK2 axis to improve ccRCC treatment.

## Introduction

In the United States, kidney cancer ranks sixth among men and ninth among women in estimated new cancer cases by sex in 2022, causing approximately 8960 deaths in men and 4960 deaths in women [1]. Renal cell carcinoma (RCC) is the most common form of kidney cancer, accounting for up to 85% of all cases, and clear cell RCC (ccRCC) is the most common subtype of RCC, occurring in 70% to 75% of all cases [2]. Over the past decade, medical therapy for RCC has shifted from non-specific immune approaches (in the era of cytokines) to targeted therapy against vascular endothelial growth factor (VEGF), and now to novel immunotherapy agents [3, 4]. Although the five-year relative survival rate at diagnosis has improved to some extent, the overall prognosis remains poor, especially for patients with high-stage tumor [5]. Therefore, it is of great significance to continuously explore new mechanisms regulating the occurrence and development of renal cancer and to find new therapeutic targets to inhibit the progression of advanced renal cancer.

Zinc finger protein 582 (ZNF582), a member of the zinc finger protein family, functions as a transcription factor. Previous reports have found the hypermethylation of ZNF582 in a variety of tumors, including cervical neoplasm [6], esophageal squamous cell carcinoma [7], oral cancer [8], nasopharyngeal carcinoma [9]. Hypermethylated ZNF582 is associated with the progression and poor prognosis of multiple tumors. For instance, hypermethylated ZNF582 is associated with aggressive progression and poor prognosis of oral cancer [10]; ZNF582 hypermethylation promotes metastasis of nasopharyngeal carcinoma by regulating the transcription of adhesion molecules Nectin-3 and NRXN3 [9]; ZNF582 promoter methylation can predict cervical cancer radiosensitivity and ZNF582 protein overexpression reduces tumor radiosensitivity [11]. Moreover, quantitative detection of ZNF582 methylation contributes to the diagnosis of clinical cervical cancer [12].

Although there have been some studies on ZNF582 in tumors, the function and regulation mechanism of ZNF582 are still less studied. At present, there are few studies on ZNF582 in renal cancer. Recently, only one study pointed out that decreased expression of ZNF582 mediated by DNA methylation is related to the progression of ccRCC [13], but the evidence is not particularly sufficient and the mechanism of ZNF582 in ccRCC has not been explored. Therefore, the methylation status, expression pattern, biological function and regulatory mechanism of ZNF582 in ccRCC deserve further exploration.

In this study, we identified the low expression pattern and hypermethylation status of ZNF582 in ccRCC, and the negative regulatory effect of DNA methylation on ZNF582 expression is also clarified. Moreover, we elucidated a new mechanism by which ZNF582 overexpression up-regulates TJP2 protein expression, which leads to the enhancement of combination of TJP2 and ERK2 protein, up-regulation of ERK2 expression level and down-regulation of ERK2 phosphorylation level, therefore suppressing the growth and metastasis of ccRCC.

## Materials and methods

### Ethics statement

This study is approved by the ethics committee of Ministry of Science and Technology of the People’s Republic of China (Approval no. 2021SLCJ2189, 2021.09.14, Beijing, China). Informed consent signed by each patient has been obtained in this study.

### Bioinformatic data mining

The mRNA expression data of ZNF582, TJP2 and ERK2 are obtained from five Gene Expression Omnibus (GEO) datasets (GSE40435, GSE66272, GSE105261, GSE126964 and GSE73731) and TCGA-KIRC (The Cancer Genome Atlas-Kidney Renal Clear Cell Carcinoma) dataset. DNA methylation data of ZNF582 is obtained from TCGA-KIRC dataset. The clinicopathological and survival data of these patients are also obtained.

### Clinical samples collection

Paraffin section samples of 60 patients diagnosed with ccRCC are provided by the Department of Urology, Peking University First Hospital. Clinicopathological information of these patients are also obtained.

### Cell culture

The normal human renal tubular epithelial cell line HK2 and five ccRCC cell lines OSRC2, 786-O, Caki-1, 769-P and A498 are used in the study, and these cell lines are cultured according to conditions specified by the provider. All cell lines are authenticated and are verified to be mycoplasma negative. ZNF582 overexpression (with 3x Flag) and TJP2 overexpression (with 3x Flag) plasmids and TJP2 knockdown plasmid (short hairpin RNA, shRNA) are constructed.

First, 293 T cells are transfected with the corresponding vector using Lipofectamine 3000 Transfection Reagent (Invitrogen, USA) according to the manufacturer’s instructions. After 48 h, cells transfected with the corresponding vector are harvested for Western Blot to observe the overexpression or knockdown efficiency. After verifying the overexpression or knockdown efficiency of the target genes in 293 T cells, the stably transfected ccRCC cell lines are established by lenti-virus infection. Lenti-virus is produced using three vectors system, and the cells are infected by lentiviruses according to the MOI value (the number of lentiviruses per number of cells). Finally, the stably transfected cell lines are screened with corresponding antibiotics, and Western Blot is also used to test the overexpression or knockdown efficiency.

### Immunohistochemistry

Immunohistochemistry is used to detect the protein expression of ZNF582, TJP2 and ERK2 in 60 pairs of ccRCC and adjacent normal renal (AN) tissues and the protein expression of E-cadherin and N-cadherin in the lung metastases of mice kidney tumors. The details of these antibodies are as follows: anti-ZNF582 (1:300, ab254814, Abcam), anti-TJP2 (1:200, 18900-1-AP, Proteintech), anti-ERK2 (1:100, ab32081, Abcam), anti-E-cadherin (1:500, ab40772, Abcam) and anti-N-cadherin (1:1000, ab19348, Abcam).

### Western Blot

Total protein of ccRCC cells and tissues is obtained using RIPA lysis buffer containing protease inhibitors, and BCA protein assay Kit is used to quantitate the protein levels. Then, Western Blot is used to detect the protein expression of ZNF582, TJP2, ERK2, p-ERK2, MEK1/2, p-MEK1/2, BCL-2, Caspase-3, Cleaved Caspase-3, E-cadherin and N-cadherin in these samples. The details of these antibodies were as follows: anti-ZNF582 (1:1000, SAB1408372, Sigma), anti-Flag (1:1000, 14793 S, CST), anti-TJP2 (1:1000, 18900-1-AP, Proteintech), anti-ERK2 (1:1000, ab32081, Abcam), anti-ERK1 (pT202/pY204) + ERK2 (pT185/pY187) (1:5000, ab76299, Abcam), anti-MEK1 + MEK2 (1:20000, ab178876, Abcam), anti-MEK1 + MEK2 (phospho S217 + S221) (1:5000, ab278723, Abcam), anti-BCL-2 (1:2000, 12789-1-AP, Proteintech), anti-Caspase-3 (1:1000, 9662 S, CST), anti-Cleaved Caspase-3 (1:1000, AF7022, Affinity), anti-E-cadherin (1:10000, ab40772, Abcam), anti-N-cadherin (1:1000, 9664 S, CST) and anti-GAPDH (1:8000, 10494-1-AP, Proteintech).

### Massarray DNA methylation assay

One: Extraction of qualified genomic DNA from ccRCC cells; Two: The DNA sample to be tested is treated with the NaHSO3 kit, so that the unmethylated cytosine (C) undergoes deamination reaction and is converted into uracil (U), while the methylated cytosine cannot undergo deamination reaction and remains as cytosine. Three: PCR amplification reaction, the PCR mixtures are pre-heated for 4 min at 94 °C, followed by 45 cycles of 94 °C for 20 s, 56 °C for 30 s and 72 °C for 1 min, the final extension at 72 °C for 3 min, and the PCR primers sequences are as follows: Forward: aggaagagag AGGTATTTAAGGGTATTTTTGGTGG; Reverse: cagtaatacgactcactatagggagaaggct CCTATAATCCCAACATTTTAAAAAACC. Four: SAP digestion reaction: the PCR product is treated with SAP (shrimp alkaline phosphatase, shrimp alkaline phosphatase) to remove free dNTPs. Five: Transcriptase cleavage reaction; Six: Resin purification; Seven: Chip spotting and EpiTYPER™ Analysis and data export.

### Demethylation analysis

OSRC2 and Caki-1 cells are seeded in six-well plates and treated with five μM 5-Aza-2′-deoxycytidine (5-Aza-dC, A, Sigma-Aldrich) for four days. Besides, cells are cultured with or without 100 Nm Trichostatin A (TSA, T, Sigma-Aldrich) for the final 24 h. Then DNA is isolated for Massarray DNA methylation assay and protein is extracted for Western Blot.

### Cell proliferation assay

Cell proliferation is determined by an ethynyl-2-deoxyuridine (EdU) incorporation assay using an EdU Apollo DNA in vitro kit (RiboBio, Guangzhou, China) and BeyoClick™ EdU Cell Proliferation Kit with DAB (Beyotime, China). For cell clone formation experiment, 200 OSRC2 and Caki-1 cells are seeded in six-well plates. After culturing for two to three weeks, stain with 0.5% crystal violet for 10 min and count the number of clones under a microscope.

### Cell apoptosis assay

One Step TUNEL Apoptosis Assay Kit (Beyotime, China) is used to detect cell apoptosis in cell slide. Colorimetric TUNEL Apoptosis Assay Kit (Beyotime, China) is used to detect cell apoptosis in the paraffin sections of mice kidney tumors.

### Wound healing assay

Wound healing assay is used to determine the cell migration ability. Briefly, approximately 1 × 10^6^ cells are seeded in six-well plates at equal densities and grow to 85% ~95% confluency. Then, artificial gaps are generated by a one ml sterile pipette tip after transfection with the corresponding lentivirus. Wounded areas are marked and photographed under a microscope at 0 h and 48 h.

### Cell transwell migratory and invasive assays

For cell transwell migration assay, 1 × 10^4^ OSRC2 and 5 × 10^3^ Caki-1 cells are plated into the upper chambers (24-well insert, pore size eight μm, Corning) with 100 μL serum-free DMEM, respectively. The lower chambers are filled with 600 μL DMEM containing 15% FBS. 36 h later, cells under the surface of the lower chamber are washed with PBS and stained with 0.5% crystal violet for 20 min. For cell invasion assay, 2 × 10^4^ OSRC2 and 1 × 10^4^ Caki-1cells are seeded on upper chambers coated with 100 μL Matrigel (one:eight dilution in PBS, Corning), respectively. The culture conditions are the same as described for the transwell migration assay. After 36 h, adherent cells on the lower surface are stained with 0.5% crystal violet. The number of cells on the lower surface is photographed with a microscope.

### TMT (Tandem mass tags) quantitative proteomics test

After extracting the total protein of cell samples and quantifying the protein concentration with BCA method, the protein samples are precipitated with acetone. Protein precipitates are redissolved, disulfide bonds are reduced with dithiothreitol, and the reduced disulfide bonds are alkylated with iodoacetamide. Mix trypsin with the samples in the proportion of trypsin:protein = 1:50 to hydrolyze the protein. Trypsin is mixed with protein samples in a ratio of trypsin:protein = 1:5 to digest protein. Each sample is labeled with TMT Isobaric Label Reagent Set (Thermo Fisher Scientific, Rockford, USA), and each group of labeling samples is mixed equally. TFA is used to fully precipitate SDC in the mixed sample to remove the SDC and obtain the labeled polypeptide samples. The obtained polypeptide samples are desalted, lyophilized polypeptide samples are redissolved with mobile phase A, and then separated by RPUPLC under alkaline conditions. Next, for each sample, 2ug of total peptides are separated and analyzed with a nano-UPLC (EASY-nLC1200) coupled to a Q Exactive HFX Orbitrap instrument (Thermo Fisher Scientific) with a nano-electrospray ion source. Data dependent acquisition (DDA) is performed in profile and positive mode with Orbitrap analyzer. The original data files are searched and analyzed using Proteome Discoverer software (PD) (Thermo Fisher Scientific) and the built-in Request HT search engine.

### Co-IP (Co-immunoprecipitation)

Co-IP is used to find other proteins that might bind to the ZNF582 protein and IP is used to verify the binding between ZNF582 and target proteins. Briefly, ccRCC cells are lysed using IP lysis buffer (Beyotime, China). Anti-Flag M2 magnetic beads (Sigma, USA) are added into the supernatant of cell lysates and incubated overnight at four °C. To detect ERK2 protein binding to MEK1/2 protein, anti-MEK1/2 antibody is added into the supernatant of cell lysates and incubated overnight at four °C, and the subsequent incubation of complexes is done with protein A/G magnetic beads (Sigma, USA) for two hours at room temperature. At last, the Co-IP products are harvested after being washed three times with lysis buffer and analyzed by silver-staining, mass spectrometry and western blot.

### Orthotopic tumor growth model

Twenty five-week old B-NDG severe immunodeficiency mice (Biocytogen Pharmaceuticals (Beijing) Co., Ltd) are used for xenograft studies. The mice are randomly divided into four groups, five in each group. Approximately 10 × 10^5^ viable OSRC2 cells are resuspended in 20 μl fresh PBS and injected orthotopically into the right kidney of each mouse. Bioluminescence imaging is performed as described previously [14]. EdU (50 mg/kg) is injected intraperitoneally two-four hours before the mice are sacrificed. After killing the mice, lung ex vivo imaging is performed immediately to examine tumor metastasis. All procedures are approved by the Institutional Animal Care and Use Committee at Peking University First Hospital. No blinding is done for animal studies.

### Statistical analyses

Non-parametric Mann–Whitney test is used to detect differences in continuous variables, and result data are represented as mean ± SEM. Survival curves for patients are plotted using the Kaplan–Meier method, with log-rank tests for statistical significance. The correlation between the expression of genes are examined using Pearson’s correlation analysis. All statistical tests are two-sided, and a *P* value of <0.05 is regarded as statistical difference.

## Results

### Reduced ZNF582 expression is associated with adverse pathology and poor prognosis in ccRCC

**Fig. 1:**
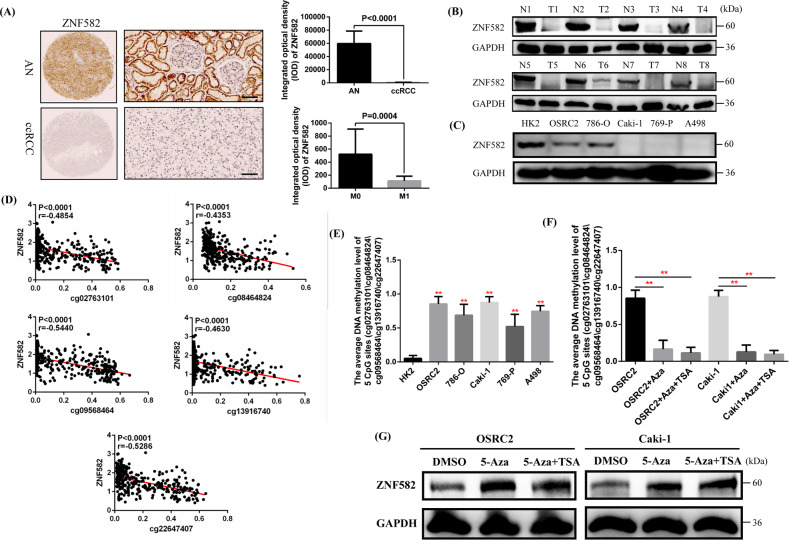
ZNF582 expression is underexpressed in ccRCC and negatively regulated by DNA methylation. **A** Comparison of ZNF582 protein expression in the paraffin sections of 60 pairs of ccRCC and AN (adjacent normal renal) tissue using Immunohistochemistry. IOD: integrated optical density. Bar: 25 um (400X). **B** Comparison of ZNF582 protein expression in eight pairs of fresh ccRCC and AN tissue using Western Blot (N: adjacent normal renal tissue, T: tumor tissue). **C** Detection of ZNF582 protein expression in five ccRCC cell lines (OSRC2, 786-O, Caki-1, 769-P and A498) and a relative control cell line (HK2) using Western Blot. **D** Correlation between ZNF582 mRNA expression and the methylation level of its five CpG sites based on TCGA-KIRC data. **E** The average methylation levels of these five CpG sites in OSRC2, 786-O, Caki-1, 769-P, A498 and HK2 cell lines using Massarray DNA methylation assay; **P < 0.01, vs. HK2. **F** The average methylation levels of these five CpG sites in OSRC2 and Caki-1 cells after treatment with 5-aza-dC and TSA demethylated ZNF582. **G** The protein expression level of ZNF582 in OSRC2 and Caki-1 cells after treatment with 5-aza-dC and TSA demethylated ZNF582. All result data are represented as mean ± SEM. **P < 0.01, compared with the corresponding control.

First, we obtained ZNF582 mRNA expression data of ccRCC patients from TCGA-KIRC dataset, and analysis results indicated that ZNF582 expression in ccRCC is significantly lower than that in the adjacent normal renal (AN) tissue (Fig. S1A). Then, we gained the ZNF582 mRNA expression data of ccRCC patients from four GEO datasets (GSE40435, GSE66272, GSE105261 and GSE126964), and analysis results also showed the expression of ZNF582 is significantly reduced in ccRCC compared with the AN tissue (Fig. S1B). Through combining the clinicopathological data of ccRCC patients in TCGA-KIRC, it was found that the expression level of ZNF582 mRNA in T3/T4, Stage III/IV, G3/G4, N1 and M1 patients is significantly decreased than that in T1/T2, Stage I/II, G1/G2, N0 and M0 patients, respectively (Fig. S1C). By combining the clinicopathological data of ccRCC patients in GSE73731, analysis results also showed that ZNF582 expression in G3/G4 ccRCC is lower than that of G1/G2 ccRCC (Fig. S1D). Moreover, it was found that ZNF582 low expression group has shorter OS (Overall Survival) and RFS (Recurrence-free Survival) than ZNF582 high expression group through combining the prognostic information of ccRCC patients in TCGA-KIRC (Fig. S1E). In addition, we examined ZNF582 protein expression in local 60 pairs of ccRCC and AN tissue by Immunohistochemistry and eight pairs of fresh ccRCC and AN tissue using Western Blot. Our results identified the low expression status of ZNF582 protein in ccRCC, and ZNF582 expression in metastatic ccRCC is lower than that of non-metastatic ccRCC (Fig. 1A, B). Finally, we detected ZNF582 protein expression in several ccRCC cell lines, and results also proved that ZNF582 is generally low expressed in ccRCC cells (OSRC2, 786-O, Caki-1, 769-P and A498) compared with the normal human renal tubular epithelial cell line HK2 (Fig. 1C). Taking the above results together, it can be concluded that ZNF582 expression is significantly decreased in ccRCC, and decreased ZNF582 expression is associated with higher tumor stage and grade, distant metastasis and poor prognosis of ccRCC patients.

### ZNF582 expression is negatively regulated by DNA methylation

Our previous study found that promoter hypermethylation is an important cause of the low expression of lncRNA ZNF582-AS1 in ccRCC [14]. Promoter of ZNF582-AS1 is a bidirectional promoter and also codes for ZNF582. Therefore, we speculated that DNA hypermethylation may also cause the decrease of ZNF582 expression in ccRCC.

We obtained the methylation data of twelve CpG sites (cg02763101, cg07135042, cg07778983, cg08464824, cg09568464, cg11740878, cg13916740, cg20984085, cg22647407, cg24039631, cg24733179 and cg25267765) of ZNF582 DNA from TCGA-KIRC. Our analysis results showed that the methylation levels of these CpG sites in ccRCC are significantly higher than those in AN tissue (Fig. S2A, B). By combining the prognostic information of these patients, survival analysis results indicated that elevated methylation levels of ten CpG sites (cg02763101, cg07135042, cg08464824, cg09568464, cg13916740, cg20984085, cg22647407, cg24039631, cg24733179 and cg25267765) are associated with shorter OS, and elevated methylation levels of five CpG sites (cg02763101, cg08464824, cg09568464, cg13916740 and cg22647407) are also associated with shorter RFS (Fig. S3A, B).

Among these CpG sites, the methylation levels of five CpG sites (cg02763101, cg08464824, cg09568464, cg13916740 and cg22647407) are significantly negatively correlated with the expression of ZNF582 mRNA (Fig. 1D). Then, we tested the methylation levels of these five CpG sites in HK2, OSRC2, 786-O, Caki-1, 769-P and A498 cell lines using Massarray DNA methylation assay, and our results confirmed that the average methylation levels of these five CpG sites in OSRC2, 786-O, Caki-1, 769-P and A498 cells are remarkably increased compared with HK2 cells (Fig. 1E). In addition, 5-Aza-2′-deoxycytidine (5-aza-dC, 5-Aza, A) and Trichostatin A (TSA, T) are used to induce the demethylation of ZNF582 DNA in OSRC2 and Caki-1 cells, and the results indicated that the methylation levels of these five CpG sites are significantly decreased after the treatment of 5-aza-dC and TSA (Fig. 1F). Western Blot results also proved that ZNF582 protein expression is remarkably increased after the treatment of 5-aza-dC and TSA (Fig. 1G). Thus, the above results demonstrated that DNA hypermethylation can lead to the decreased expression of ZNF582 protein in ccRCC.

### ZNF582 protein overexpression promotes cell apoptosis and suppresses cell proliferation, migration and invasion in vitro

**Fig. 2:**
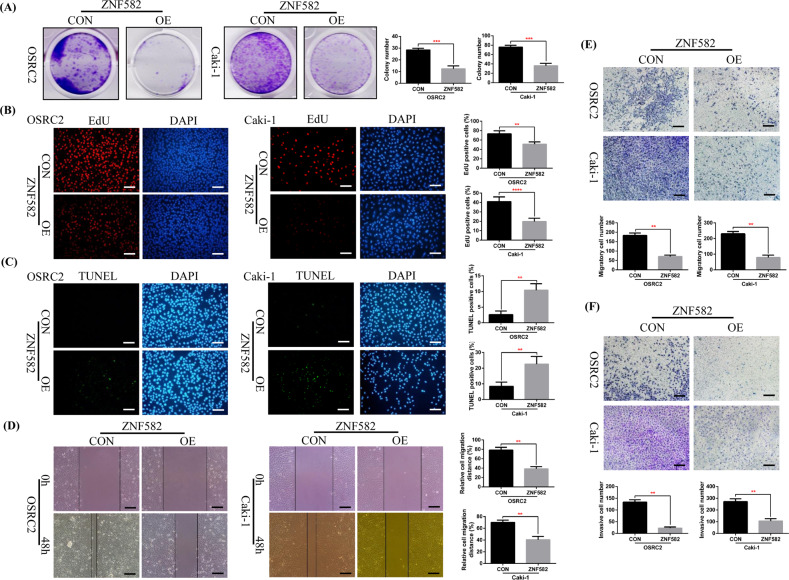
ZNF582 overexpression inhibits the growth and migration of ccRCC cell in vitro. **A** Comparison of the number of cell clones in ZNF582 overexpression and control OSRC2 and Caki-1 cells. Comparison of the proportion of EdU (**B**) and TUNEL (**C**) positive cells in ZNF582 overexpression and control OSRC2 and Caki-1 cells by immunofluorescence (200X, Bar: 50 um). **D** Wound healing assay determined the migratory distances of ZNF582 overexpression and control OSRC2 and Caki-1 cells (100X, Bar: 100 um). Comparison of cell migration (**E**) and cell invasion (**F**) ability in ZNF582 overexpression and control OSRC2 and Caki-1 cells (100X, Bar: 100 um). The presentation of the histogram was the result of three independent experiments. **P < 0.01, ***P < 0.001, **** *P* < 0.0001, compared with the corresponding control.

To clarify the effect of ZNF582 protein on ccRCC cell phenotype, we constructed ZNF582 stably transfected OSRC2 and Caki-1 cell lines. Immunofluorescence EdU method and clone formation experiment are used to detect the effect of ZNF582 overexpression on ccRCC cell growth. Immunofluorescence TUNEL method is used to detect the effect of ZNF582 overexpression on ccRCC cell apoptosis. Wound healing assay and cell migration and invasion experiments are used to detect the effect of ZNF582 overexpression on the metastatic ability of ccRCC cells. Our results verified that ZNF582 overexpression significantly suppresses growth and promotes apoptosis of OSRC2 and Caki-1 cells (Fig. 2A–C). Moreover, compared with control cells, the number of OSRC2 and Caki-1 cells that migrated and invaded after ZNF582 overexpression is also significantly reduced (Fig. 2D–F).

### ZNF582 protein binds to TJP2 and ERK2 proteins and regulates their expression

**Fig. 3:**
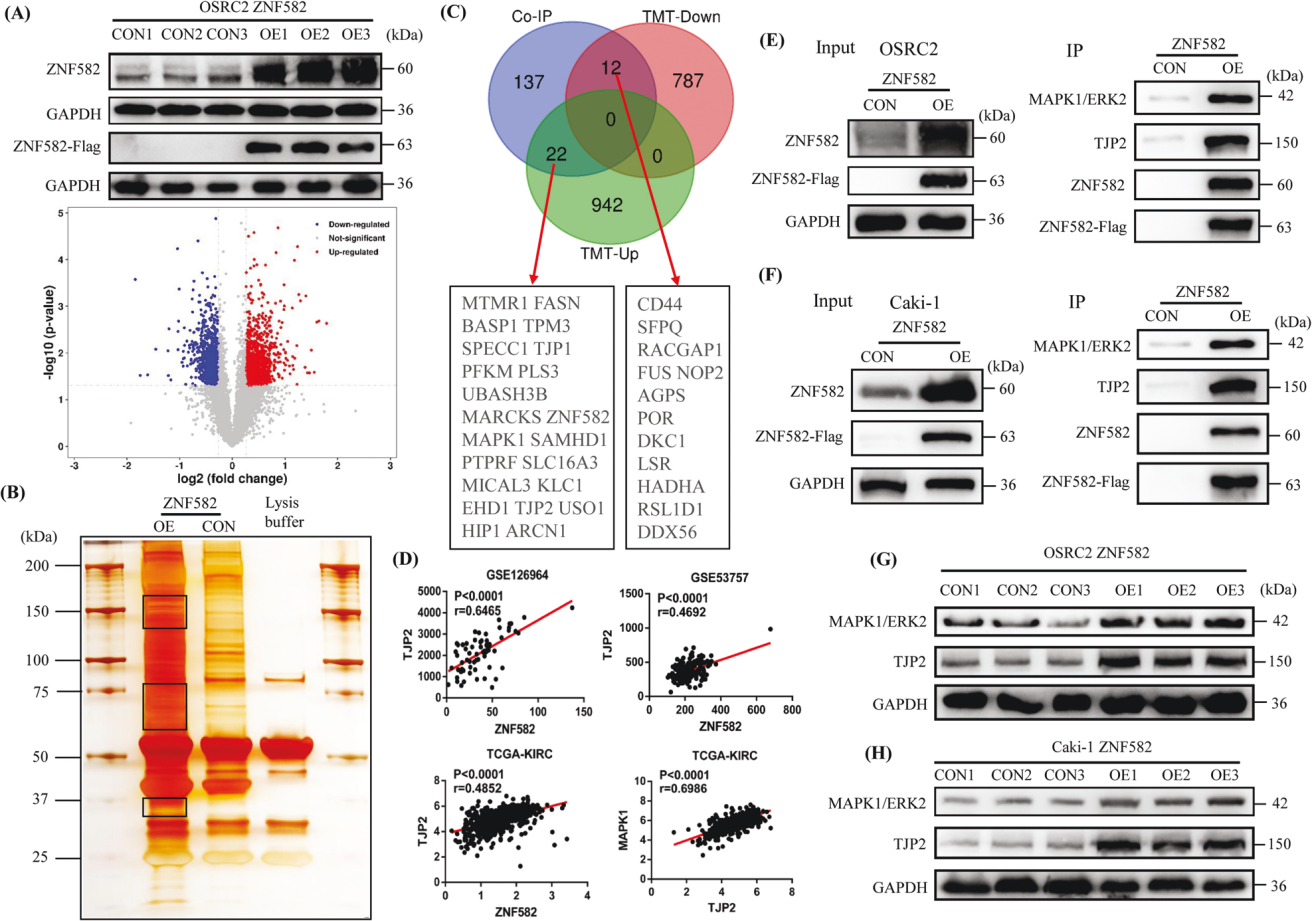
ZNF582 protein binds to TJP2 and ERK2 protein and regulates their expression. **A** Comparison of the differential protein between ZNF582 overexpression and control OSRC2 cells by TMT experiment. **B** Identification of the possible binding proteins of ZNF582 protein by Co-IP, silver-staining and mass spectrometry. **C** Venn diagram analysis of TMT results and mass spectrometry results. **D** TJP2 expression is significantly positively correlated with ZNF582 expression based on GSE126964, GSE53757 and TCGA-KIRC data, and MAPK1 (ERK2) expression is significantly positively correlated with TJP2 expression based on TCGA-KIRC data. **E**, **F** Confirmation of ZNF582 protein binds to TJP2 and ERK2 protein in OSRC2 and Caki-1 cells by IP and Western Blot. **G**, **H** Comparison of TJP2 and ERK2 expression between ZNF582 overexpression and control OSRC2 and Caki-1 cells.

In order to explore the potential mechanism by which ZNF582 overexpression inhibits the growth and metastasis of ccRCC cells, TMT quantitative proteomics method was used to detect the differentially expressed proteins between ZNF582 overexpression OSRC2 cells and control cells. TMT data analysis results showed that 964 proteins and 799 proteins are up-regulated and down-regulated in ZNF582 overexpression OSRC2 cells compare with control cells, respectively (Fig. 3A). Co-IP experiment was used to identify proteins that may bind to ZNF582, and silver-staining method was utilized to detect the differential protein bands between ZNF582 overexpression group and the control group. Compared with control group, we can clearly identify multiple different protein bands in ZNF582 overexpression group (Fig. 3B). Then, these different protein bands were sent to mass spectrometry test to detect possible proteins in them, and the test results indicate that a total of 171 proteins were detected. The combined analysis of TMT data and mass spectrometry data suggested that, compared with control group, there are 34 proteins that may be regulated by ZNF582 overexpression, including 22 up-regulated and twelve down-regulated proteins, and they have the potential to bind to ZNF582 protein (Fig. 3C). Besides, we used GSE126964, GSE53757 and TCGA-KIRC data to analyze the correlation between ZNF582 expression and the expression of other 21 genes, and analysis results indicated that only the linear correlation intensity between ZNF582 and TJP2 expression ranks the top five in these three datasets (Fig. S4). ZNF582 expression is significantly positively correlated with the expression of TJP2. In addition, we also found that TJP2 expression is significantly positively correlated with the expression of MAPK1 (also known as ERK2) (Fig. 3D). Thus, we speculate that TJP2 and ERK2 may be downstream regulatory targets of ZNF582. Then, we used anti-Flag M2 magnetic beads (Sigma) to precipitate ZNF582 protein. Anti-ZNF582 (Sigma), anti-Flag (CST), anti-TJP2 (Proteintech) and anti-ERK2 (Abcam) antibodies were used for subsequent Western Blot detection. Consistent with the hypothesis, in the same IP protein samples in OSRC2 and Caki-1 cells, TJP2 and ERK2 protein can only be detected in ZNF582 overexpression group (Fig. 3E, F). Western Blot results also identified that ZNF582 overexpression can lead to the increased expression of TJP2 and ERK2 proteins in OSRC2 and Caki-1 cells (Fig. 3G, H).

### ZNF582 overexpression up-regulates ERK2 expression by regulating TJP2

**Fig. 4:**
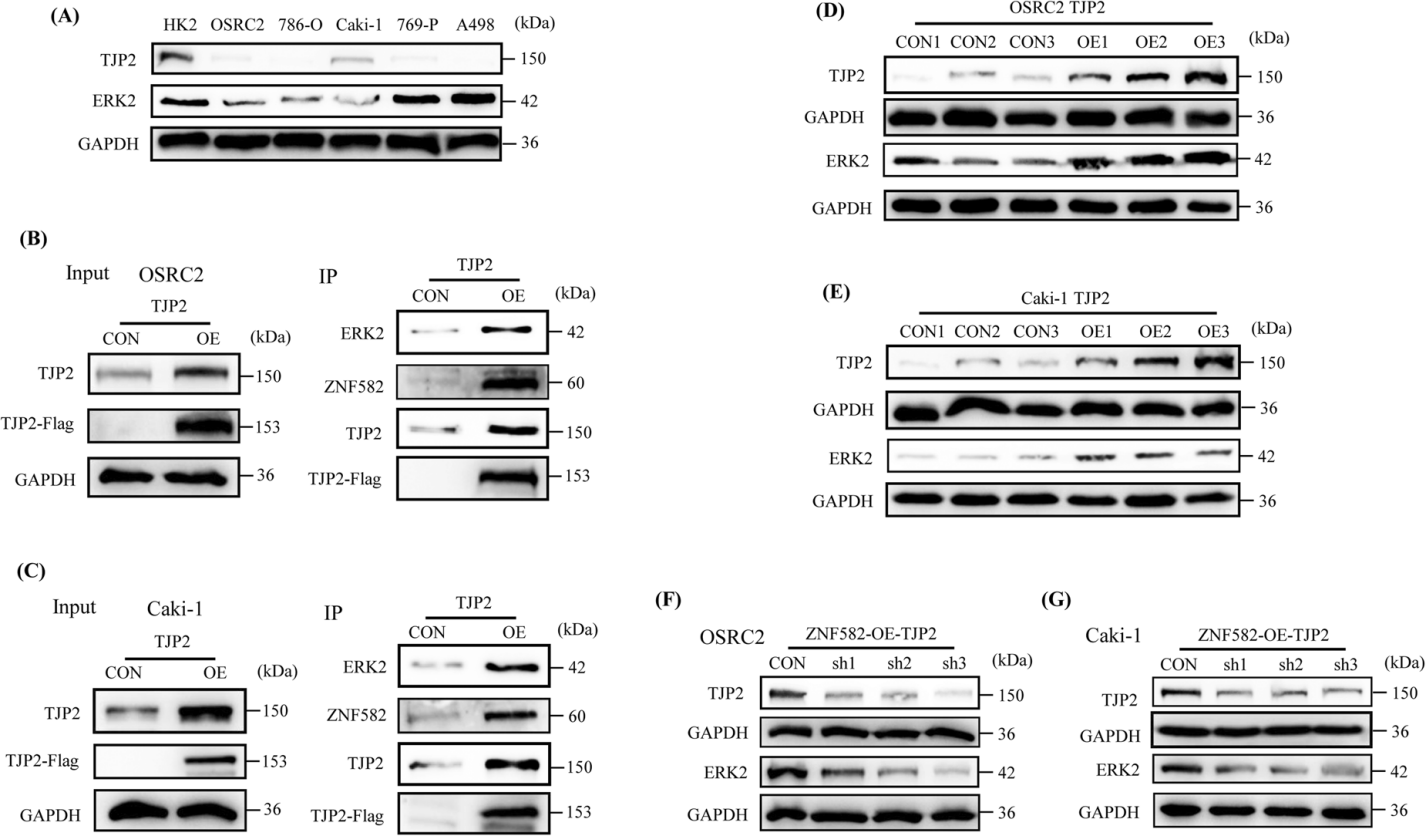
ZNF582 affects ERK2 expression by regulating TJP2. **A** Detection of TJP2 and ERK2 protein expression in OSRC2, 786-O, Caki-1, 769-P, A498 and HK2 cells. **B**, **C** Confirmation of TJP2 protein binds to ZNF582 and ERK2 protein in OSRC2 and Caki-1 cells by IP and Western Blot. **D**, **E** Comparison of ERK2 expression between TJP2 overexpression and control OSRC2 and Caki-1 cells. **F**, **G** Comparison of ERK2 expression between TJP2 knockdown and its control OSRC2 and Caki-1 cells.

TJP2 (also known as ZO-2) is a cytoplasmic protein of tight junctions (TJs), which can combine with a variety of nuclear proteins, plays the role of a transcription inhibitor, and causes inhibition of cell proliferation and transformation [15]. So, can ZNF582 directly regulate ERK2 or affect ERK2 by regulating TJP2? In order to fully understand the regulatory relationship between ZNF582 and TJP2 and ERK2, we first detected TJP2 and ERK2 protein expression in several ccRCC cell lines. Results showed that TJP2 expression is generally reduced in ccRCC cell lines and ERK2 expression is also decreased in OSRC2 and Caki-1 cells (Fig. 4A). Then, we constructed TJP2 stably transfected OSRC2 and Caki-1 cell lines. Anti-Flag M2 magnetic beads were also used to precipitate TJP2 protein, and anti-TJP2, anti-Flag, anti-ZNF582 and anti-ERK2 antibodies were also used for subsequent Western Blot detection. IP and Western Blot experiment results also indicated that TJP2 can bind to ZNF582 and ERK2 (Fig. 4B, C). Besides, ERK2 expression is also significantly increased after TJP2 overexpression in OSRC2 and Caki-1 cells (Fig. 4D, E). To further verify the regulatory effect of TJP2 on ERK2, we constructed TJP2 knockdown shRNA (short hairpin RNA). First, we verified the knockdown efficiency of TJP2 in 293 T cells. Our results showed that the knockdown efficiency of TJP2 protein increases significantly after the quality of TJP2 shRNA increased from 1ug to 4ug, and the knockdown efficiency of TJP2 protein does not change significantly after the quality of TJP2 shRNA increased to 4ug or more (Fig. S5A). Moreover, the knockdown efficiency of TJP2 protein begins to appear after 36 h of cell culture, and no obvious change is observed in the knockdown efficiency of TJP2 protein after 48 h or longer of cell culture (Fig. S5B). Therefore, no obvious dose and time response of TJP2 shRNA knockdown efficiency has been observed at present. In addition, we also successfully constructed TJP2 stable knockdown OSRC2 and Caki-1 cell lines based on the overexpression of ZNF582. After knocking down TJP2 expression, ERK2 expression in OSRC2 and Caki-1 cell lines is also decreased (Fig. 4F, G). The above results demonstrated that ZNF582 overexpression affects the expression of ERK2 by regulating TJP2.

### Binding of TJP2 and ERK2 inhibits ERK2 phosphorylation

**Fig. 5:**
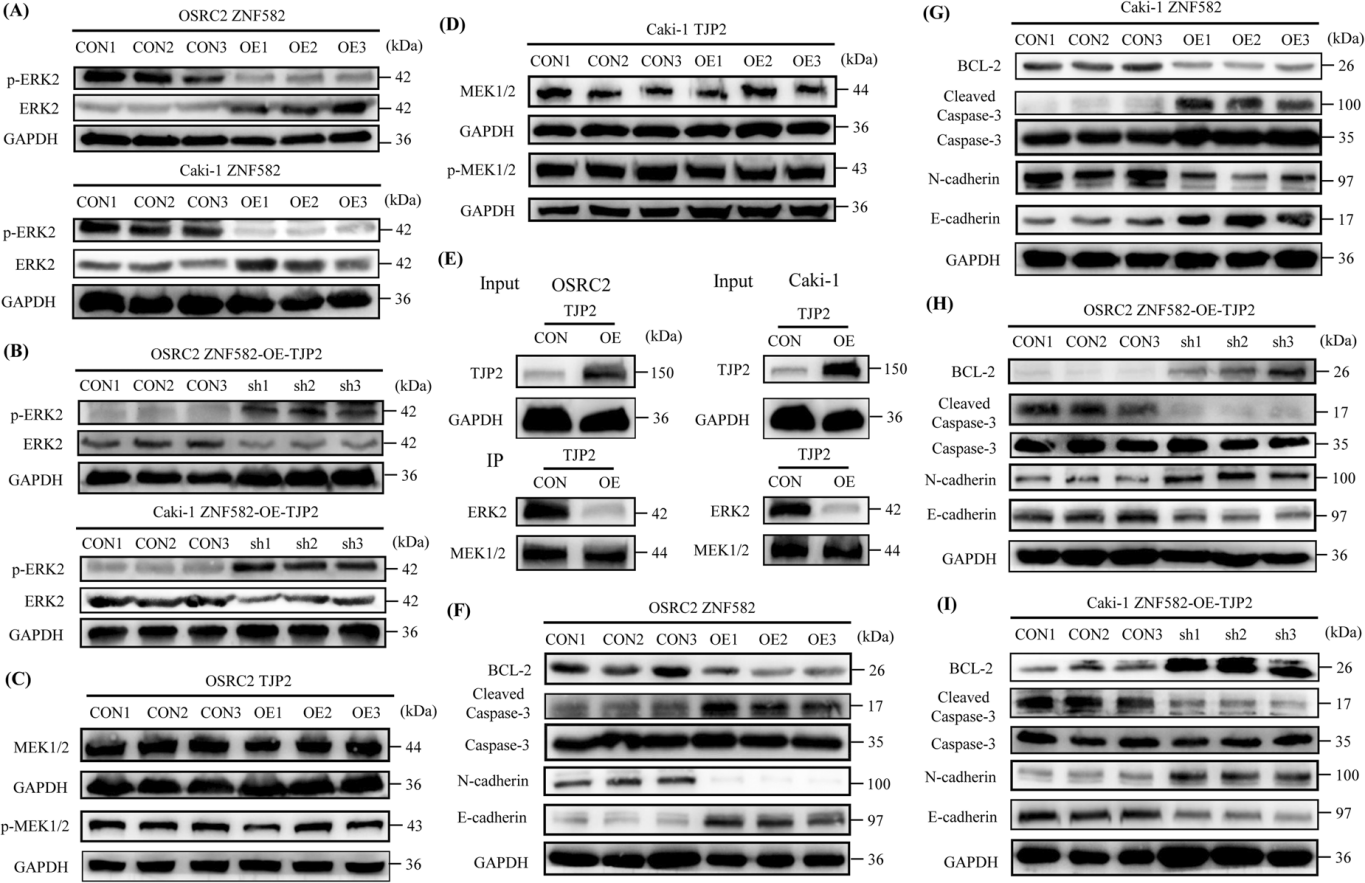
Combination of TJP2 and ERK2 inhibits ERK2 phosphorylation. **A** Detection of p-ERK2 protein expression in ZNF582 overexpression and control OSRC2 and Caki-1 cells. **B** Detection of p-ERK2 protein expression in TJP2 knockdown and control OSRC2 and Caki-1 cells. **C**, **D** Detection of MEK1/2 and p-MEK1/2 protein expression in TJP2 overexpression and control OSRC2 and Caki-1 cells. **E** Detection of the expression of ERK2 binding to MEK1/2 in TJP2 overexpression and control OSRC2 and Caki-1 cells. **F**, **G** Comparison of the expression of BCL-2, Cleaved Caspase-3, N-cadherin and E-cadherin between ZNF582 overexpression and control OSRC2 and Caki-1 cells. **H**, **I** Comparison of the expression of BCL-2, Cleaved Caspase-3, N-cadherin and E-cadherin between TJP2 knockdown and control OSRC2 and Caki-1 cells.

Like for many other protein kinases, ERK activation is mediated by phosphorylation of residues Thr185 and Tyr187 caused by MEK1/2, and activated ERK can phosphorylate a large number of substrates involved in cell proliferation, survival, growth, metabolism, migration and differentiation [16]. Therefore, we wondered whether the change of TJP2 expression will also affect the phosphorylation level of ERK2. For this reason, we detected the expression level of p-ERK2 protein in OSRC2 and Caki-1 cells with ZNF582 overexpression and TJP2 knockdown, respectively. Results indicated that p-ERK2 expression level is significantly decreased after ZNF582 overexpression, while the level of p-ERK2 protein is recovered after knockdown of TJP2 expression (Fig. 5A, B). However, we examined the expression levels of MEK1/2 and p-MEK1/2 protein in TJP2 overexpression OSRC2 and Caki-1 cells, and results showed that there is no change in the expression of MEK1/2 and p-MEK1/2 (Fig. 5C, D). Then, we used anti-MEK1/2 (Abcam) antibody and protein A/G magnetic beads (Sigma) to precipitate MEK1/2 protein, and anti-MEK1/2, anti-TJP2 and anti-ERK2 antibodies were used for subsequent Western Blot detection. Interestingly, our IP results showed that after overexpression of TJP2, the ERK2 protein binds to MEK1/2 protein is significantly reduced (Fig. 5E), which suggests that the combination of TJP2 and ERK2 may hinder the phosphorylation of ERK2 caused by MEK1/2.

In addition, we detected the expression of proteins related to cell apoptosis, proliferation and metastasis in OSRC2 and Caki-1 cells with ZNF582 overexpression and TJP2 knockdown, respectively. Results displayed that the protein expression of Cleaved Caspase-3 and E-cadherin is significantly increased and the protein expression of BCL-2 and N-cadherin was significantly decreased after ZNF582 overexpression (Fig. 5F, G). Furthermore, on the basis of ZNF582 overexpression, knockdown of TJP2 expression can reverse the increase in Cleaved Caspase-3 and E-cadherin expression and the decrease in BCL-2 and N-cadherin expression (Fig. 5H, I). Taken together, we demonstrated that ZNF582 overexpression can up-regulate ERK2 expression and down-regulate p-ERK2 expression by regulating TJP2, thereby causing the increase in Cleaved Caspase-3 and E-cadherin expression and the decrease in BCL-2 and N-cadherin expression.

### Reduced TJP2 and ERK2 expression is correlated with adverse pathology and poor prognosis in ccRCC

**Fig. 6:**
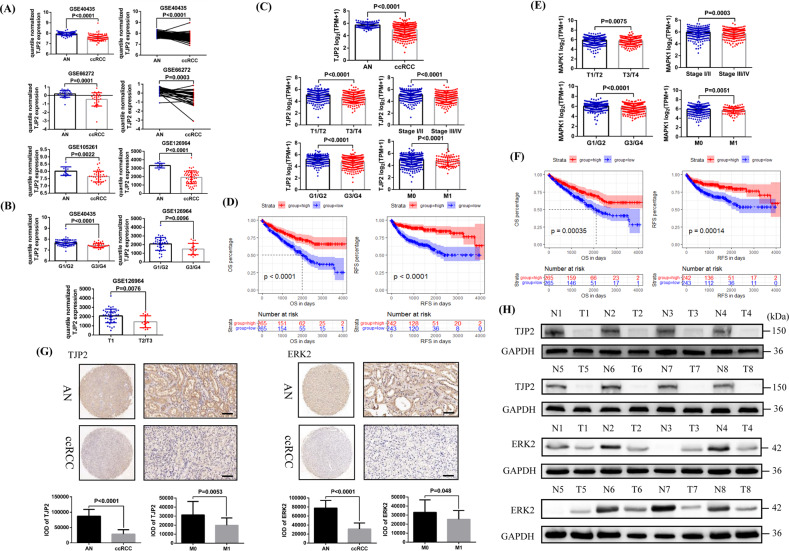
TJP2 and ERK2 are lowly expressed in ccRCC and reduced TJP2 and ERK2 expression is associated with poor pathology and prognosis. **A** Comparison of TJP2 mRNA expression in ccRCC and AN (adjacent normal renal) tissue based on GSE40435, GSE66272, GSE105261 and GSE126964 data. **B** Comparison of TJP2 mRNA expression in G1/G2 and G3/G4 patients, T1 and T2/T3 patients based on GSE40435 and GSE126964 data. **C** Comparison of TJP2 mRNA expression in ccRCC and AN tissue, T1/T2 and T3/T4 patients, Stage I/II and Stage III/IV patients, G1/G2 and G3/G4 patients, and M0 and M1 patients based on TCGA-KIRC data. **D** Comparison of the prognostic differences between TJP2 mRNA high expression and low expression groups in TCGA-KIRC patients. **E** Comparison of ERK2 mRNA expression in T1/T2 and T3/T4 patients, Stage I/II and Stage III/IV patients, G1/G2 and G3/G4 patients, and M0 and M1 patients based on TCGA-KIRC data. **F** Comparison of the prognostic differences between ERK2 mRNA high expression and low expression groups in TCGA-KIRC patients. **G** Comparison of TJP2 and ERK2 protein expression in the paraffin sections of 60 pairs of ccRCC and AN tissue using Immunohistochemistry. IOD: integrated optical density. Bar: 25 um (400X). **H** Comparison of TJP2 and ERK2 protein expression in eight pairs of fresh ccRCC and AN tissue using Western Blot (N: adjacent normal renal tissue, T: tumor tissue). All result data are represented as mean ± SEM.

In the above, we have clarified the mechanism by which ZNF582 overexpression up-regulates ERK2 by regulating TJP2, and then the clinical value of TJP2 and ERK2 also need to be identified. By analyzing the data of GEO datasets (GSE40435, GSE66272, GSE105261 and GSE126964), results showed that TJP2 is significantly down-regulated in ccRCC compared with the AN tissue (Fig. 6A). Decreased TJP2 expression is associated with higher tumor grade and stage (Fig. 6B). By analyzing TCGA-KIRC data, results also indicated that TJP2 is also significantly decreased in ccRCC and decreased TJP2 expression is associated with higher tumor grade and stage, distant metastasis and poor prognosis (Fig. 6C, D). As for ERK2, reduced ERK2 expression is also found to be associated with higher tumor grade and stage, distant metastasis and poor prognosis by analyzing TCGA data (Fig. 6E, F). In addition, we verified the low expression status of TJP2 and ERK2 in ccRCC and their relationship with ccRCC metastasis by immunohistochemical staining in 60 pairs of ccRCC and AN tissue and Western Blot in eight pairs of fresh ccRCC and AN tissue (Fig. 6G, H).

### Knockdown of TJP2 expression reverses the phenotype inhibition of ccRCC cells caused by ZNF582 overexpression in vitro and in vivo

**Fig. 7:**
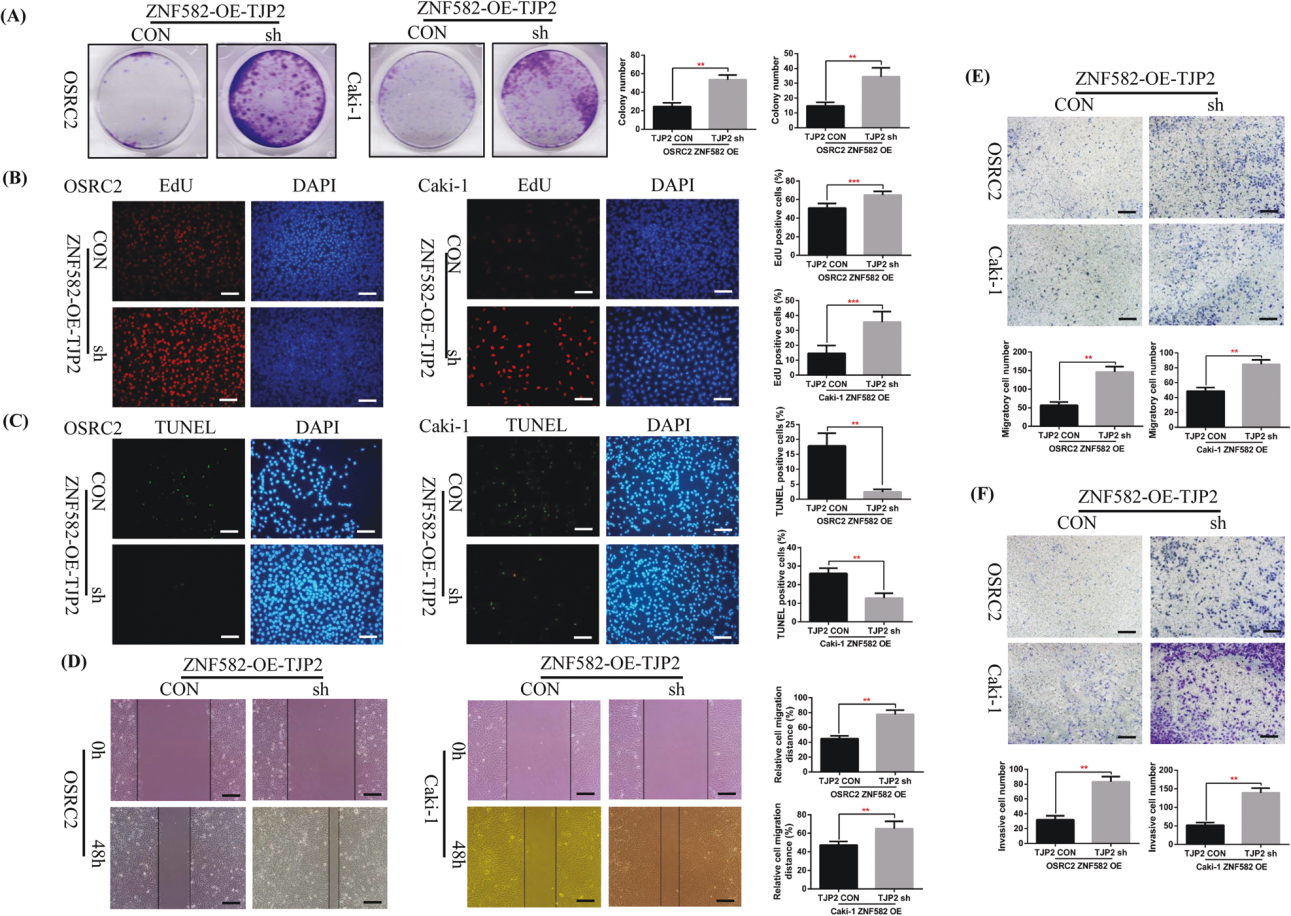
Knockdown of TJP2 expression reverses the phenotype inhibition of ccRCC cell caused by ZNF582 overexpression in vitro. **A** Comparison of the number of cell clones in TJP2 knockdown and control OSRC2 and Caki-1 cells. Comparison of the proportion of EdU (**B**) and TUNEL (**C**) positive cells in TJP2 knockdown and control OSRC2 and Caki-1 cells by immunofluorescence (200X, Bar: 50 um). **D** Wound healing assay determined the migratory distances of TJP2 knockdown and control OSRC2 and Caki-1 cells (100X, Bar: 100 um). Comparison of cell migration (**E**) and cell invasion (**F**) ability in TJP2 knockdown and control OSRC2 and Caki-1 cells (100X, Bar: 100 um). The presentation of the histogram was the result of three independent experiments. **P < 0.01, ***P < 0.001, compared with the corresponding control.

The above results have determined that knockdown of TJP2 can reverse the increase of Cleaved Caspase-3 and E-cadherin levels and the decrease of BCL-2 and N-cadherin levels caused by ZNF582 overexpression, the question is whether knockdown of TJP2 expression can also reverse the phenotype inhibition caused by ZNF582 overexpression. Thus, we constructed TJP2 stably knockdown OSRC2 and Caki-1 cell lines based on ZNF582 overexpression. Our results proved that after knocking down the expression of TJP2 based on ZNF582 overexpression, cell proliferation is significantly increased and cell apoptosis is significantly decreased (Fig. 7A–C). Besides, cell migration and invasion ability is remarkably restored (Fig. 7D–F).

**Fig. 8:**
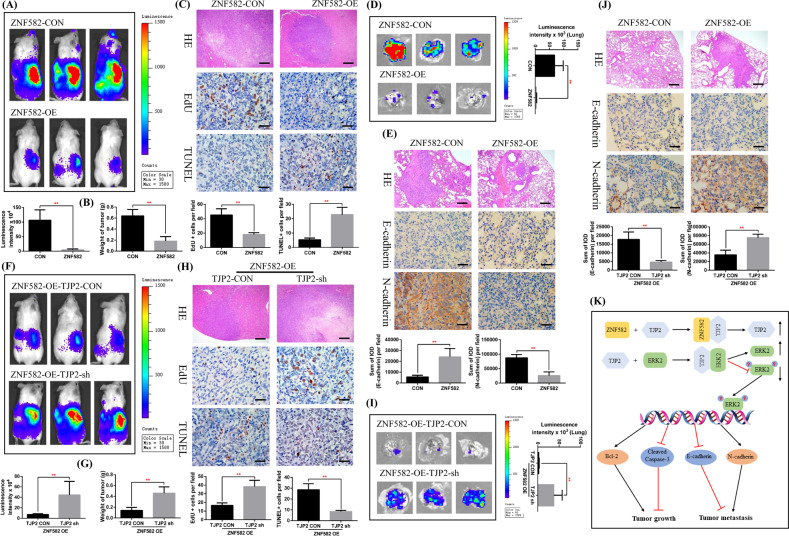
ZNF582 overexpression inhibits ccRCC cell growth and metastasis by regulating TJP2 in vivo. **A** Representative bioluminescence imaging of ZNF582 overexpression and control groups and quantification of these bioluminescence imaging. **B** Quantification of the tumor weight of ZNF582 overexpression and control groups. **C** Representative picture of HE (100X, Bar: 100 um), EdU and TUNEL expression (400X, Bar: 25 um) in the kidney tumors of ZNF582 overexpression and control groups and quantification the expression of EdU and TUNEL. **D** Representative lung ex vivo bioluminescence imaging of ZNF582 overexpression and control groups and quantification of ex vivo imaging. **E** Representative picture of HE (100X, Bar: 100 um), E-cadherin and N-cadherin expression (400X, Bar: 25 um) in the lung metastases of ZNF582 overexpression and control groups and quantification of these picture. IOD: integrated optical density. **F** Representative bioluminescence imaging of TJP2 knockdown and control groups and quantification of these bioluminescence imaging. **G** Quantification of the tumor weight of TJP2 knockdown and control groups. **H** Representative picture of HE (100X, Bar: 100 um), EdU and TUNEL expression (400X, Bar: 25 um) in the kidney tumors of TJP2 knockdown and control groups and quantification the expression of EdU and TUNEL. **I** Representative lung ex vivo bioluminescence imaging of TJP2 knockdown and control groups and quantification of ex vivo imaging. **J** Representative picture of HE (100X, Bar: 100 um), E-cadherin and N-cadherin expression (400X, Bar: 25 um) in the lung metastases of TJP2 knockdown and control groups and quantification of these picture. IOD: integrated optical density. **K** Schematic model of ZNF582 overexpression up-regulates TJP2 protein expression, which leads to up-regulation of ERK2 protein and down-regulation of p-ERK2, therefore suppressing the growth and metastasis of ccRCC. All result data are represented as mean ± SEM.

To verify the inhibitory effect of ZNF582 on ccRCC tumor growth and metastasis in vivo, we constructed a mouse orthotopic tumor growth model. Either ZNF582 overexpression or control OSRC2 cells were orthotopically injected into the renal capsules of B-NDG severe immunodeficiency mice. Tumor growth in vivo was confirmed by bioluminescence imaging. Our results showed that the growth rate of ZNF582 overexpression cell is significantly slower than that in control cells in vivo (Fig. 8A, B). Besides, we used HE staining to visualize the structure of tumor tissue and Immunohistochemistry to detect the staining of EdU and TUNEL, and results indicated that EdU staining number is significantly decreased and TUNEL staining number is significantly increased in the kidney tumors of ZNF582 overexpression group compared with control group (Fig. 8C). Moreover, lung ex vivo imaging data indicated that ZNF582 overexpression also inhibits the spontaneous lung metastasis of tumor cells (Fig. 8D). HE staining was also used to visualize the structure of the lung metastases and Immunohistochemistry was used to detect the expression of E-cadherin and N-cadherin. Staining results showed that E-cadherin expression is significantly increased and N-cadherin expression is significantly decreased in the lung metastases of ZNF582 overexpression group compared with control group (Fig. 8E). Furthermore, after knocking down TJP2 expression based on ZNF582 overexpression, the tumor growth rate is significantly restored (Fig. 8F). EdU staining number is remarkably increased and TUNEL staining number is remarkably decreased (Fig. 8G, H). Meanwhile, the lung metastases are remarkably increased, E-cadherin expression is remarkably reduced and N-cadherin expression is remarkably enhanced (Fig. 8I, J).

Taken together, we firstly clarified the low expression status of ZNF582 in ccRCC and its clinical value, revealed the regulatory effect of DNA methylation on ZNF582 expression, and elucidated a new mechanism by which ZNF582 overexpression up-regulates TJP2 protein expression, which leads to up-regulation of ERK2 protein and down-regulation of ERK2 protein phosphorylation, therefore suppressing ccRCC growth and metastasis (Fig. 8K).

## Discussion

On the molecular level, ccRCC is characterized by copy number changes, such as chromosome 3 p loss and VHL inactivation [17]. Other driving mutations, including SETD2, PBRM1 and BAP1, lead to genomic instability and promote tumor cell metastasis through the disorder of various metabolic and immune response pathways [18]. Currently, mutation, gene expression and proteomic characteristics can be used for early diagnosis and prognosis prediction of ccRCC [19]. However, the application of these in the diagnosis and prognosis of ccRCC and their clinical application have not been realized.

Previous studies have shown that the expression of ZNF582 and its methylation status can be used as biomarkers for tumor biological diagnosis and prognosis prediction. For instance, methylated ZNF582 gene is used as a marker for triage of women with Pap smear reporting low-grade squamous intraepithelial lesions [20]; hypermethylated ZNF582 is an effective biomarker for detection of oral cancer [8, 21]; abnormal DNA methylation of ZNF582 gene is a potential biomarker of esophageal squamous cell carcinoma [22]; hypermethylated ZNF582 gene is associated with invasive progression and poor prognosis of oral cancer [10]; ZNF582 methylation level also can predict the radiosensitivity of cervical cancer [23].

In the study, our results identified that ZNF582 mRNA and protein expression is significantly decreased in ccRCC compared with the AN tissue by analyzing GEO and TCGA-KIRC datasets and using Immunohistochemistry and Western Blot experiments to validate in local ccRCC samples. Reduced ZNF582 expression is also closely related to higher tumor stage and grade, distant metastasis and shorter OS and RFS. Previous findings agreed that hypermethylation status of ZNF582 DNA can lead to the down-regulation of ZNF582 expression [6, 9]. Consistent with these results, we found that the DNA methylation levels of all twelve CpG sites of ZNF582 DNA are significantly higher in ccRCC than that in the AN tissue by analyzing the DNA methylation data in TCGA-KIRC dataset. Moreover, higher methylation levels of cg02763101, cg07135042, cg08464824, cg09568464, cg13916740, cg20984085, cg22647407, cg24039631, cg24733179 and cg25267765 are associated with shorter OS, while higher methylation levels of cg02763101, cg08464824, cg09568464, cg13916740 and cg22647407 are associated with shorter RFS. Furthermore, we identified the negative regulatory effect of DNA methylation on ZNF582 expression through Massarray DNA methylation assay and demethylation analysis.

Therefore, ZNF582 expression and its methylation level may be an effective marker for the diagnosis and prognosis prediction of ccRCC. In addition, based on our above findings and those of previous studies [9, 10, 12, 22, 24], we speculated that ZNF582 hypermethylation status and low expression level may be a common phenomenon in multiple tumors. In order to verify this view, it is necessary to verify the expression of ZNF582 and its methylation status in more tumor types in the near future.

Since the expression feature and clinical value of ZNF582 in ccRCC have been clarified, we next explored whether increasing ZNF582 expression has an impact on the phenotype of ccRCC cells. Using Immunofluorescence EdU and TUNEL method, clone formation experiment, wound healing assay, cell migration and invasion experiments and orthotopic kidney tumor growth model, we verified the inhibitory effect of ZNF582 overexpression on ccRCC growth and metastasis in vitro *and* in vivo. Then, the underlying mechanism of ZNF582-mediated ccRCC inhibition need to be addressed. Through TMT and Co-IP experiments, we identified two proteins (TJP2 and ERK2) that can bind to and be regulated by ZNF582 protein. Besides, by analyzing GEO and TCGA datasets and validating using local clinical ccRCC samples, it was found that both TJP2 and ERK2 are lowly expressed in ccRCC. Reduced TJP2 and ERK2 expression are also associated with worse pathology and poorer prognosis. Moreover, TJP2 expression is positively correlated with the expression of ZNF582 and ERK2, while ERK2 is only positively correlated with the expression of TJP2. However, it is unknown whether TJP2 plays a bridge role in ZNF582 regulating ERK2.

TJP2 belongs to the membrane associated guanylate kinase homologue (MAGUK) protein family and is concentrated at the cytoplasmic face of TJs in epithelial cells [25]. TJP2 is able to regulate cell proliferation [26, 27] and apoptosis [28, 29] and interact with nuclear proteins [30, 31]. Besides, several lines of evidence suggested that TJP2 expression is silenced in multiple carcinomas, including breast carcinoma [32], pancreas adenocarcinoma [33] and as well as in a hypoxia-resistant cancer cell lines derived from a scirrhous gastric carcinoma [34]. Moreover, some studies directly suggested that TJP2 plays the role of a tumor suppressor protein [35, 36].

ERK2 is part of a family of structurally related kinases, named mitogen-activated protein kinase 1 (MAPK1), whose signaling mechanism depended on an activating phosphorylation cascade that involves two upstream kinases commonly known as MAPK kinases (MAPKKs) and MAPKK kinases (MAPKKKs) [37]. ERK activation can trigger a large transcriptional programme to support cell proliferation, suppress proapoptotic factors and stimulate antiapoptotic proteins [38–40]. ERK2 can phosphorylate TOPK/PBK to promote tumorigenesis and participate in sorafenib resistance of renal cancer [41]; ERK2-induced PDHE1α phosphorylation and subcellular translocation promote tumor immune escape [42]. In this study, we found that TJP2 and ERK2 are also low expressed in ccRCC, and their decreased expression is also related to worse pathology and poor prognosis. Moreover, ZNF582 can bind to TJP2 protein and regulate its expression, and TJP2 can bind to ERK2 protein and regulate its expression. Increased binding of TJP2 to ERK2 may inhibit the phosphorylation of ERK2, thereby affecting the growth and metastasis of ccRCC.

Finally, we detected the expression of apoptosis and invasion related proteins in ZNF582 overexpression and knockdown ccRCC cells and mouse kidney tumors, our results determined that, after ZNF582 overexpression, the protein expression of Cleaved Caspase-3 and E-cadherin is significantly increased and the protein expression of BCL-2 and N-cadherin is significant decreased. Meanwhile, the increased Cleaved Caspase-3 and E-cadherin expression and the decreased BCL-2 and N-cadherin expression is reversed after knockdown of TJP2 expression based on ZNF582 overexpression. Thus, these results further proved that ZNF582 inhibits ccRCC growth and metastasis by regulating TJP2.

Zinc finger protein family is one of the most common transcription factor families in eukaryotic cells, with more than 3000 members in the human genome, and plays an important role in many biological processes [43]. They are known to play a key role in regulating expression of genes important for complicated biological processes, including cell proliferation, apoptosis, metabolism, autophagy, immune response, stem cell maintenance and differentiation [43, 44]. Due to their multiple functions, some zinc finger proteins are powerful regulators in the development of tumors, including renal cancer. For example, ZNF677 inhibits RCC progression through N6-methyladenosine and transcriptional repression of CDKN3 [45]. Therefore, in addition to the regulatory effect of ZNF582 on the growth and metastasis of ccRCC in this study, more studies are also needed to explore the regulatory effects of ZNF582 on other pathways, such as cell metabolism, autophagy, immune response, stem cell maintenance and differentiation.

In general, we elucidated a new mechanism by which ZNF582 overexpression up-regulates TJP2 protein expression, which leads to up-regulation of ERK2 protein and down-regulation of p-ERK2, therefore suppressing the growth and metastasis of ccRCC.

Our findings for the first time provided a solid theoretical basis for targeting the ZNF582/TJP2/ERK2 axis to improve ccRCC treatment. However, there are still some limitations in our research. Although we have confirmed that ZNF582 can bind to TJP2 protein and up-regulate its expression, the specific regulatory mechanism remains unclear. Similarly, although we have identified that TJP2 can bind to ERK2 protein and up-regulate its expression, the detailed regulatory mechanism has not been fully elucidated. Hence, in the near future, more studies are needed to determine the specific binding region of ZNF582 and TJP2 protein to explore the regulatory mode of ZNF582 on TJP2. The specific binding sites and binding modes of TJP2 and ERK2 protein, as well as their effects on ERK2 phosphorylation sites, also need to be further explored.

## Supplementary information

Reproducibility checklist

Final supplemental figure legends

Final supplemental figures

Original Data File

## Data Availability

The datasets generated during and/or analyzed during the current study are available from the corresponding author on reasonable request.
