# Supplementary material for: ZNF582 overexpression restrains the progression of clear cell renal cell carcinoma by enhancing the binding of TJP2 and ERK2 and inhibiting ERK2 phosphorylation
Source: Cell Death Dis. 2023 Mar 25;14(3):212. doi: 10.1038/s41419-023-05750-y (PMC10039855; doi:10.1038/s41419-023-05750-y)
Supplement: Supplementary file 2 — Final supplemental figure legends [file 41419_2023_5750_MOESM2_ESM.docx]

**Supplemental Figure 1: ZNF582 mRNA is low expressed in ccRCC, and reduced ZNF582 expression is related to worse pathology and poorer prognosis.**

**A** Comparison of ZNF582 mRNA expression in ccRCC (n=539) and adjacent normal renal (AN) tissue (n=72) based on TCGA-KIRC data. **B** Comparison of ZNF582 mRNA expression in ccRCC and AN tissue based on GSE40435 (n=101), GSE66272 (27), GSE105261 (n(AN)=nine, n(ccRCC)=35) and GSE126964 (n(AN)=nine, n(ccRCC)=55) data. **C** Comparison of ZNF582 mRNA expression in T1/T2 and T3/T4 patients, Stage I/II and Stage III/IV patients, G1/G2 and G3/G4 patients, N0 and N1 patients, and M0 and M1 patients based on TCGA-KIRC data; N0 (no lymph node invasion), N1 (with lymph node invasion), M0 (no distant metastasis), M1 (with distant metastasis). **D** Comparison of ZNF582 mRNA expression in G1/G2 and G3/G4 patients based on GSE73731 data. **E** Comparison of the prognostic differences between ZNF582 mRNA high expression group and low expression group in TCGA-KIRC patients.

**Supplemental Figure 2: The methylation levels of the CpG sites of ZNF582 are significantly elevated in ccRCC**

**A** Heatmap showing the methylation levels of twelve CpG sites of ZNF582 in ccRCC and AN tissue based on TCGA-KIRC data. **B** Comparison of the methylation levels of these twelve CpG sites in ccRCC and AN tissue.

**Supplemental Figure 3: Elevated methylation levels of the CpG sites of ZNF582 DNA are associated with poor prognosis**

**A** The relationship between methylation levels of the CpG sites of ZNF582 and OS in ccRCC patients based on TCGA-KIRC data. **B** The relationship between methylation levels of the CpG sites of ZNF582 and RFS in ccRCC patients based on TCGA-KIRC data.

**Supplemental Figure 4: The correlation between ZNF582 expression and the expression of other 21 genes based on GSE126964, GSE53757 and TCGA-KIRC data**

**Supplemental Figure 5: Detection of dose and time response of TJP2 shRNA knockdown efficiency. A** Detection of dose response of TJP2 shRNA knockdown efficiency. **B** Detection of time response of TJP2 shRNA knockdown efficiency.
