# Supplementary material for: ZNF582 overexpression restrains the progression of clear cell renal cell carcinoma by enhancing the binding of TJP2 and ERK2 and inhibiting ERK2 phosphorylation
Source: Cell Death Dis. 2023 Mar 25;14(3):212. doi: 10.1038/s41419-023-05750-y (PMC10039855; doi:10.1038/s41419-023-05750-y)
Supplement: Supplementary file 3 — Final supplemental figures [file 41419_2023_5750_MOESM3_ESM.pdf]

Supplemental Figure 1

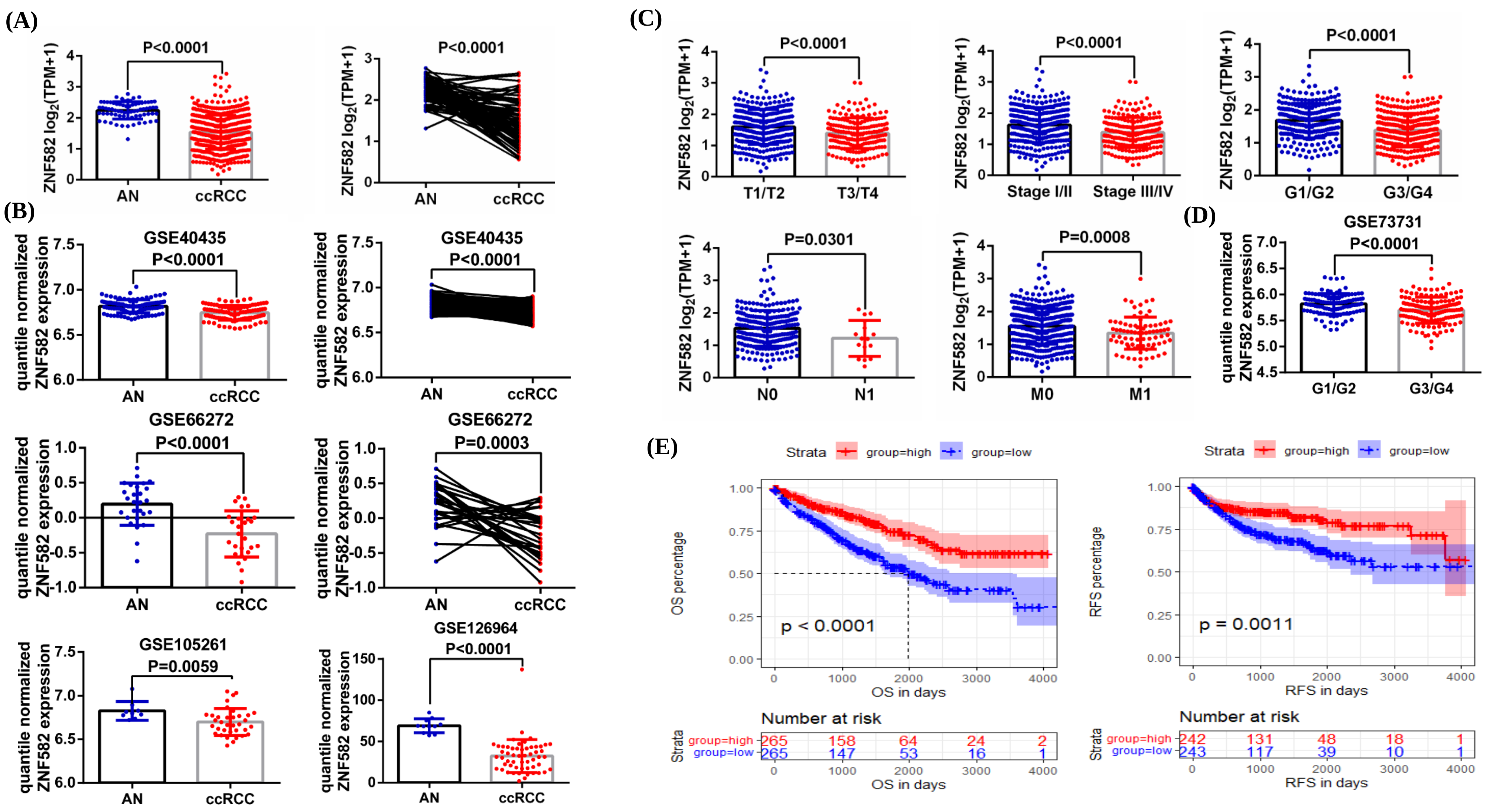

(A)

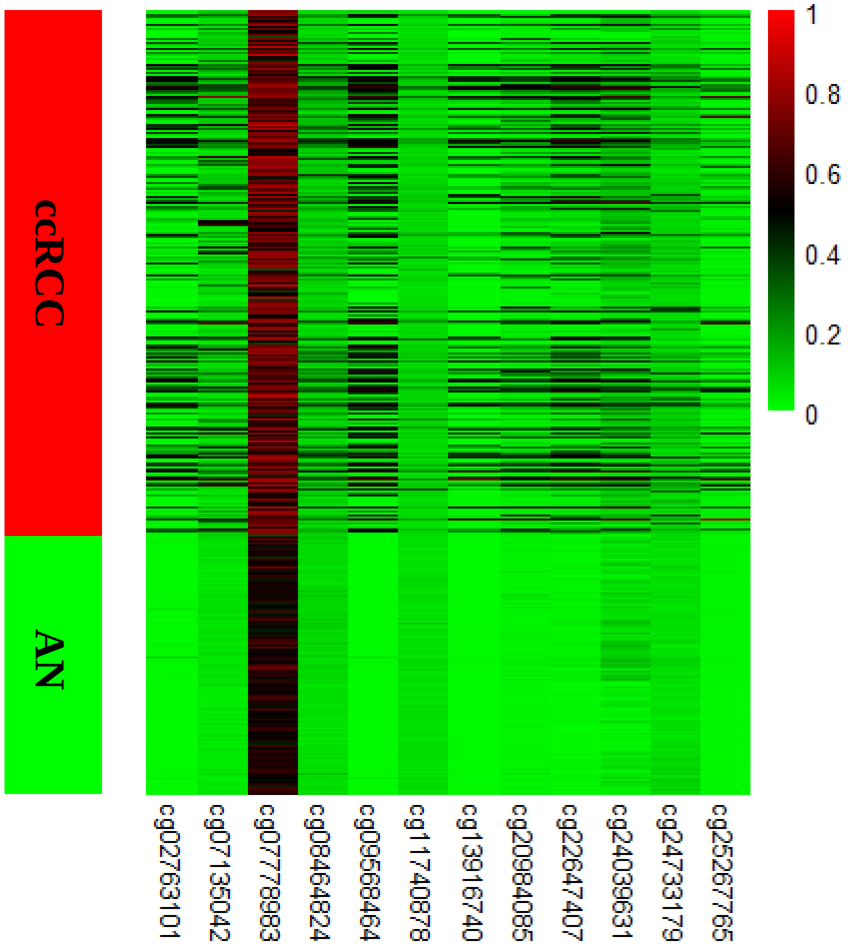
ccRCC
AN
1
0.8
0.6
0.4
0.2
0
cg02763101
cg07135042
cg07778983
cg08464824
cg09568464
cg11740878
cg13916740
cg20984085
cg22647407
cg24039631
cg24733179
cg25267765

(B)

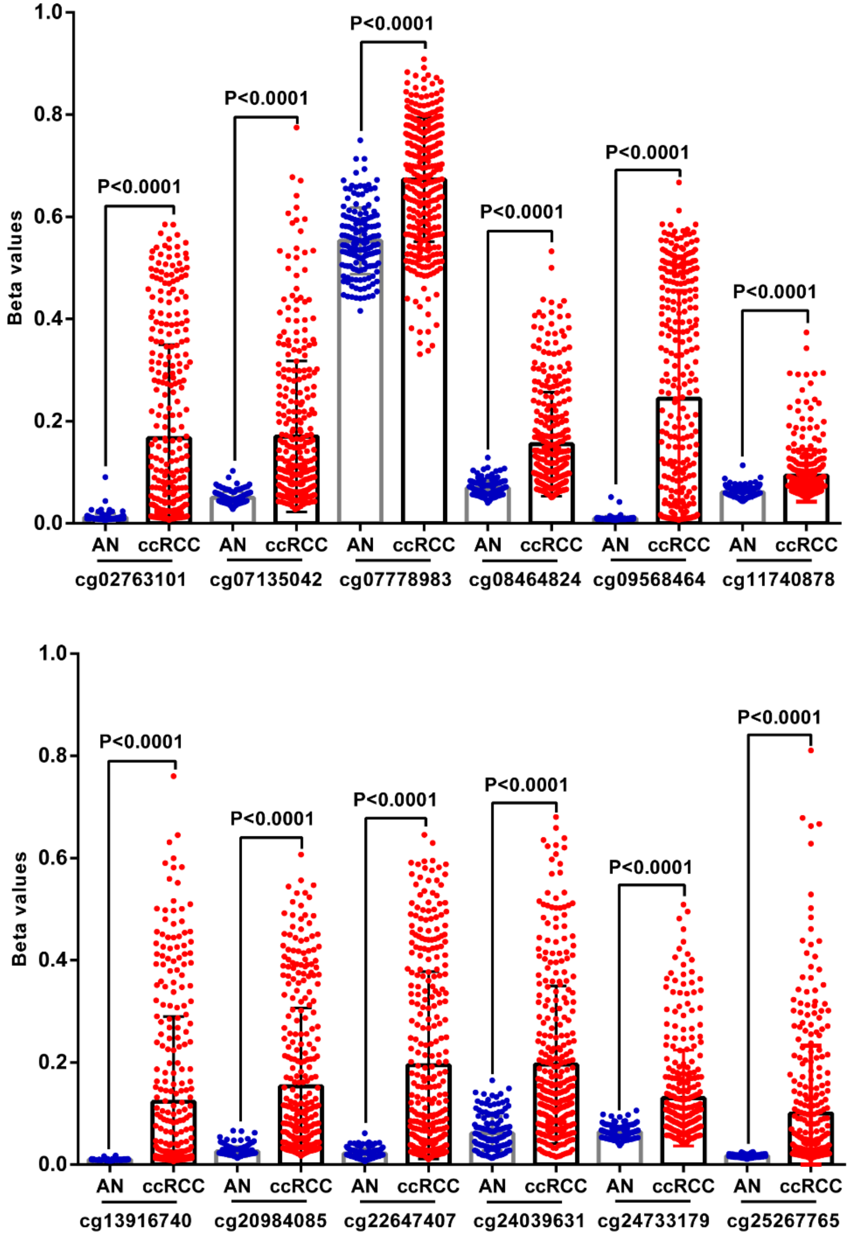
Beta values
1.0
0.8
0.6
0.4
0.2
0.0
P<0.0001
AN ccRCC
cg02763101
cg07135042
cg07778983
cg08464824
cg09568464
cg11740878
cg13916740
cg20984085
cg22647407
cg24039631
cg24733179
cg25267765

Supplemental Figure 3

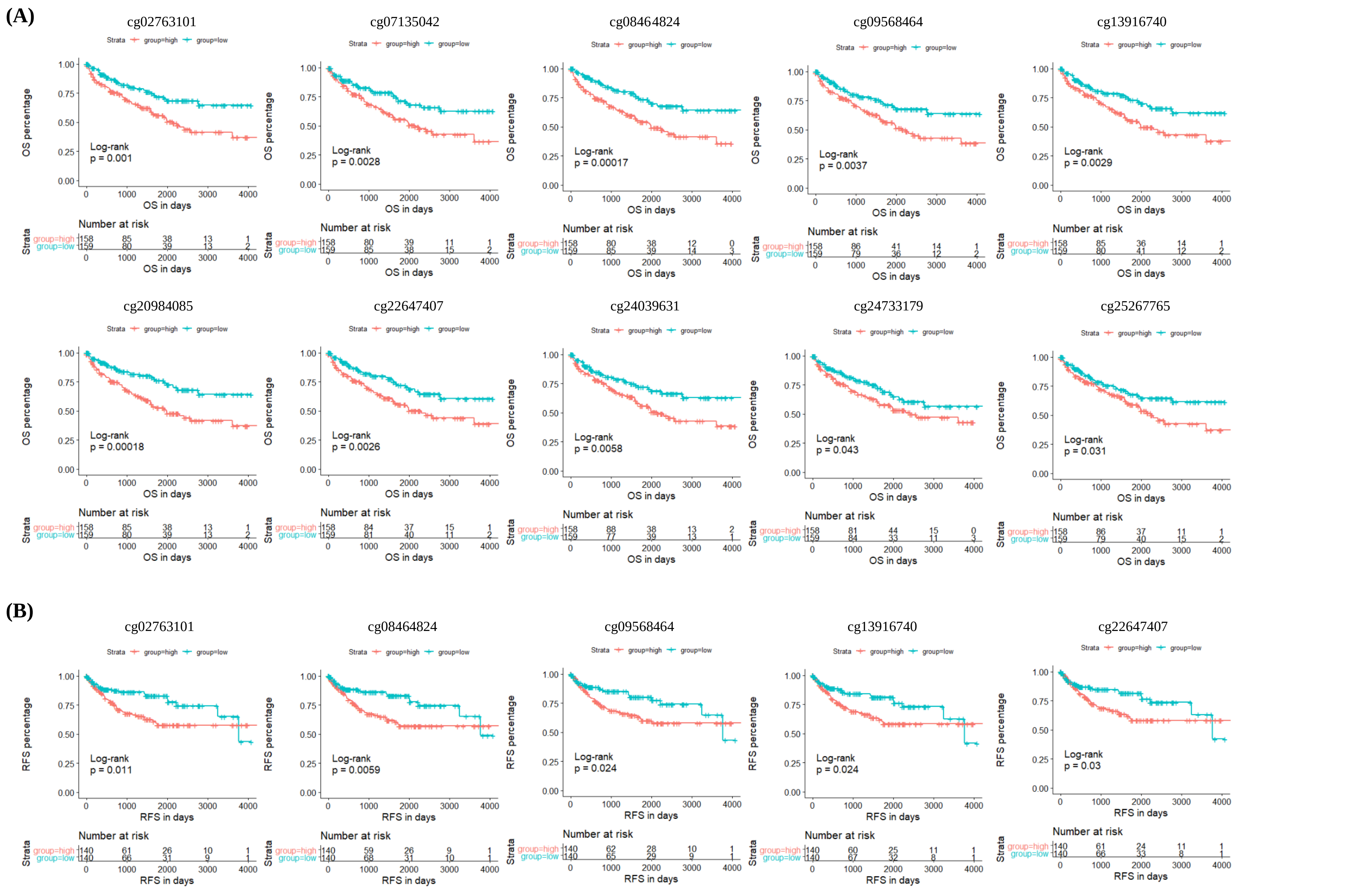

Supplemental Figure 4

GSE126964

Gene1	Gene2	P	r	|r|
ZNF582	TJP2	4.55E-09	0.646459	0.646459
ZNF582	PFKM	5.31E-09	0.644302	0.644302
ZNF582	SLC16A3	1.06E-08	-0.63457	0.634567
ZNF582	PLS3	5.45E-07	0.571326	0.571326
ZNF582	PTPRF	1.09E-05	-0.51236	0.512363
ZNF582	MARCKS	3.39E-05	-0.48687	0.48687
ZNF582	MAPK1	6.37E-05	-0.47177	0.471769
ZNF582	TPM3	2.78E-04	-0.43339	0.433394
ZNF582	SAMHD1	2.95E-04	-0.43177	0.431772
ZNF582	TJP1	5.17E-04	0.415748	0.415748
ZNF582	EHD1	0.008207	0.322793	0.322793
ZNF582	FASN	0.019771	-0.28632	0.286321
ZNF582	BASP1	0.022731	-0.28011	0.280106
ZNF582	KLC1	0.024735	0.276279	0.276279
ZNF582	MTMR1	0.084396	-0.21404	0.214043
ZNF582	UBASH3B	0.10215	-0.20297	0.202973
ZNF582	USO1	0.144413	-0.18163	0.181631
ZNF582	ARCN1	0.275976	0.136071	0.136071
ZNF582	MICAL3	0.466495	-0.09119	0.09119
ZNF582	HIP1	0.815585	-0.02926	0.029261

GSE53757

Gene1	Gene2	P	r	|r|
ZNF582	USO1	1.26E-12	-0.54734	0.547339
ZNF582	SLC16A3	6.53E-11	-0.51013	0.510135
ZNF582	PFKM	2.35E-10	0.497036	0.497036
ZNF582	TJP2	3.00E-09	0.469187	0.469187
ZNF582	MTMR1	3.37E-09	-0.46785	0.467854
ZNF582	MARCKS	6.61E-09	-0.46002	0.460015
ZNF582	SAMHD1	7.68E-09	-0.45824	0.458242
ZNF582	PTPRF	3.76E-05	-0.33633	0.336328
ZNF582	EHD1	2.03E-04	0.304834	0.304834
ZNF582	MAPK1	8.67E-04	-0.27458	0.274581
ZNF582	KLC1	9.17E-04	-0.27333	0.273332
ZNF582	ARCN1	0.005168	-0.23187	0.23187
ZNF582	BASP1	0.023438	-0.1888	0.188796
ZNF582	HIP1	0.044564	-0.16767	0.16767
ZNF582	FASN	0.072056	0.150354	0.150354
ZNF582	SPECC1	0.245398	0.097419	0.097419
ZNF582	PLS3	0.351848	0.078147	0.078147
ZNF582	MICAL3	0.60182	0.043843	0.043843
ZNF582	TPM3	0.62424	0.041162	0.041162
ZNF582	TJP1	0.753726	0.026369	0.026369
ZNF582	UBASH3B	0.922323	-0.0082	0.008197

TCGA-KIRC

Gene1	Gene2	P	r	|r|
ZNF582	TJP1	2.55E-46	0.562609	0.562609
ZNF582	PLS3	3.57E-42	0.540279	0.540279
ZNF582	SPECC1	8.87E-40	0.526566	0.526566
ZNF582	TJP2	3.49E-33	0.485234	0.485234
ZNF582	KLC1	1.33E-29	0.460172	0.460172
ZNF582	ARCN1	6.16E-22	0.398298	0.398298
ZNF582	USO1	4.08E-21	0.39084	0.39084
ZNF582	PFKM	3.75E-17	0.351844	0.351844
ZNF582	MARCKS	2.06E-16	0.34395	0.34395
ZNF582	HIP1	2.31E-16	0.34339	0.34339
ZNF582	MTMR1	5.08E-16	0.339652	0.339652
ZNF582	MAPK1	1.14E-11	0.286856	0.286856
ZNF582	UBASH3B	5.11E-11	0.277938	0.277938
ZNF582	TPM3	5.20E-07	0.214158	0.214158
ZNF582	MICAL3	1.05E-06	0.208471	0.208471
ZNF582	SAMHD1	9.85E-06	0.189117	0.189117
ZNF582	SLC16A3	2.04E-04	-0.15934	0.159336
ZNF582	BASP1	0.071664	-0.07765	0.077648
ZNF582	PTPRF	0.200531	-0.05522	0.055221
ZNF582	EHD1	0.4544	0.032289	0.032289
ZNF582	FASN	0.667364	0.018553	0.018553

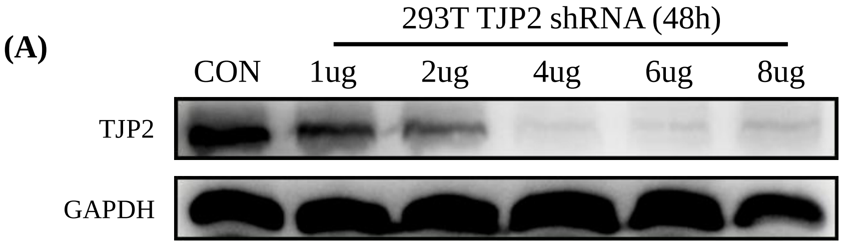
(A)
293T TJP2 shRNA (48h)
CON
1ug
2ug
4ug
6ug
8ug
TJP2
GAPDH

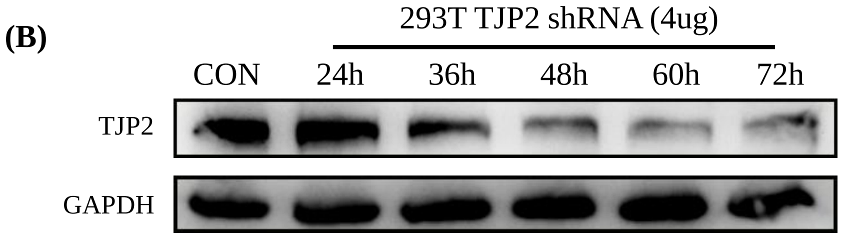
(B)
293T TJP2 shRNA (4ug)
CON
24h
36h
48h
60h
72h
TJP2
GAPDH
