# Supplementary material for: ZNF582 overexpression restrains the progression of clear cell renal cell carcinoma by enhancing the binding of TJP2 and ERK2 and inhibiting ERK2 phosphorylation
Source: Cell Death Dis. 2023 Mar 25;14(3):212. doi: 10.1038/s41419-023-05750-y (PMC10039855; doi:10.1038/s41419-023-05750-y)

Full and uncropped western blots

Figure 1B

ZNF582 N1-T4

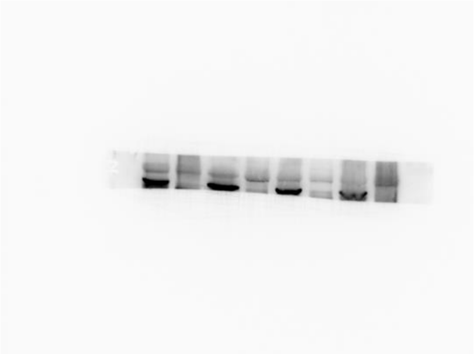

GAPDH N1-T4

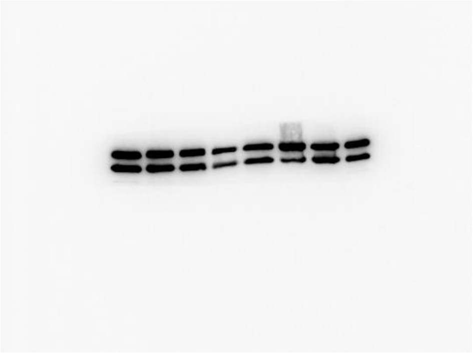

ZNF582 N5-T8

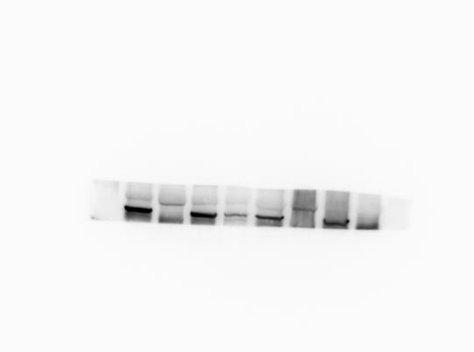

GAPDH N5-T8

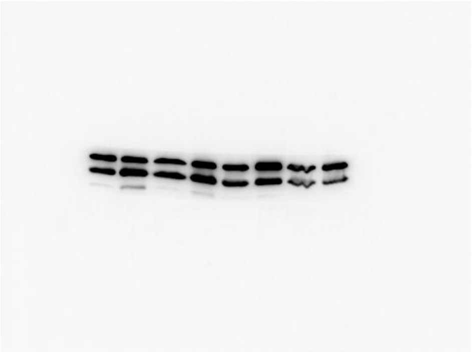

Figure 1C

ZNF582

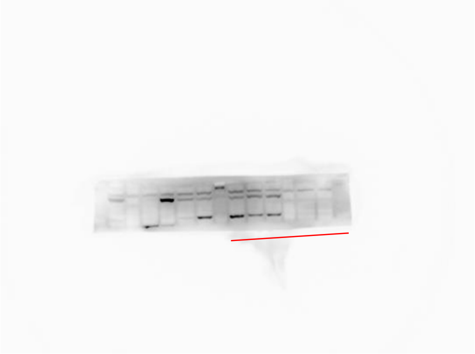

GAPDH

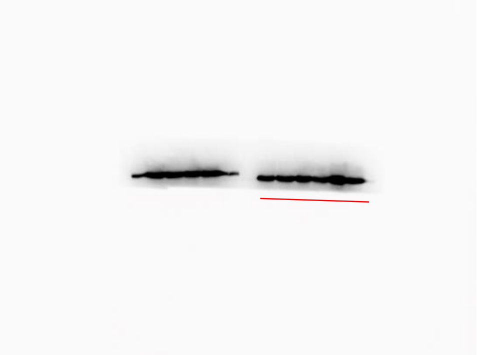

Figure 1G

ZNF582

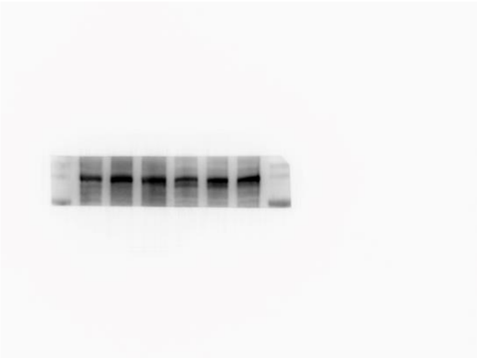

GAPDH

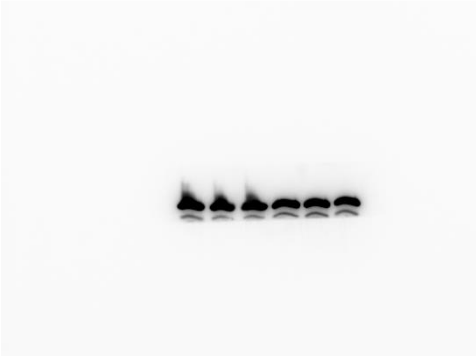

Figure 3A

ZNF582

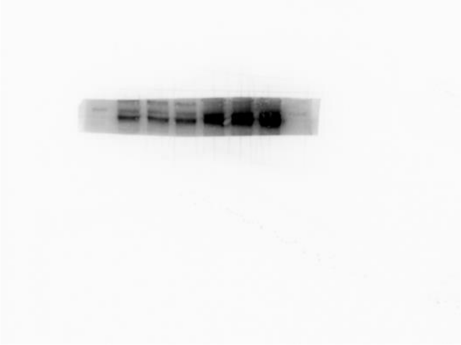

GAPDH

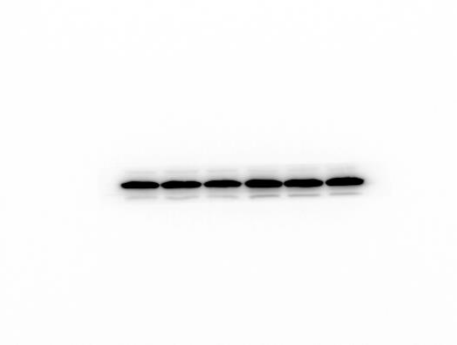

ZNF582-Flag

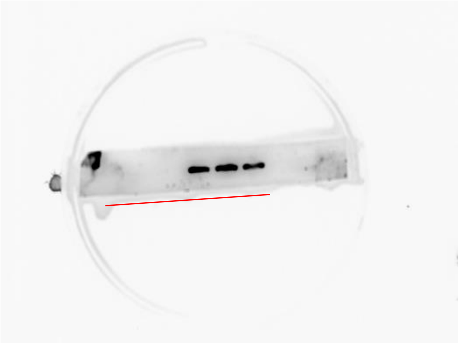

GAPDH

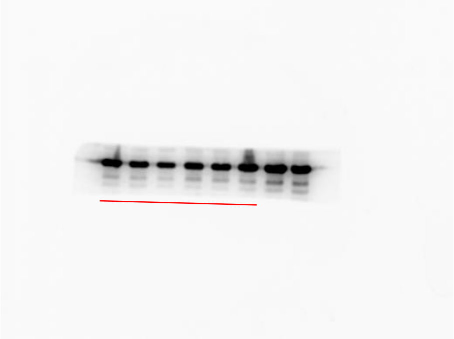

Figure 3E

Input ZNF582

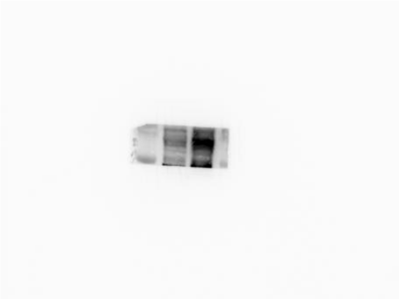

Input ZNF582-Flag

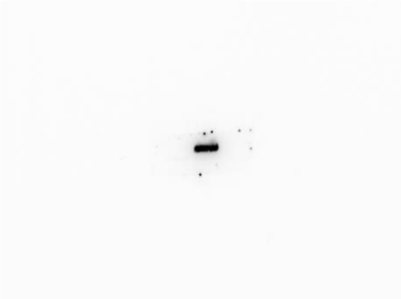

Input GAPDH

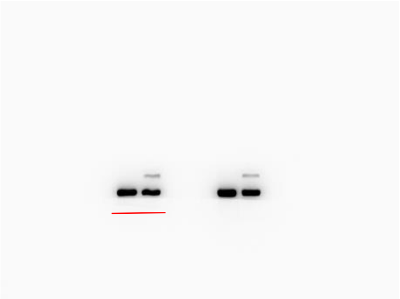

IP ERK2

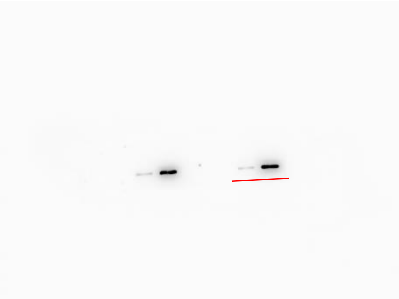

IP TJP2

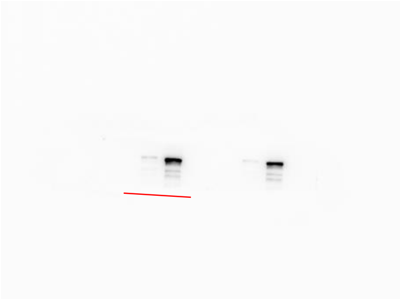

IP ZNF582

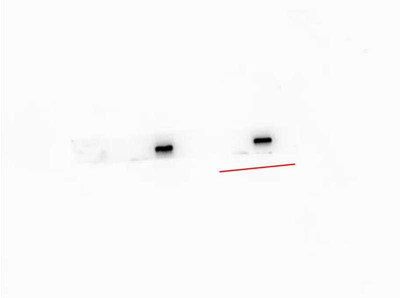

IP ZNF582-Flag

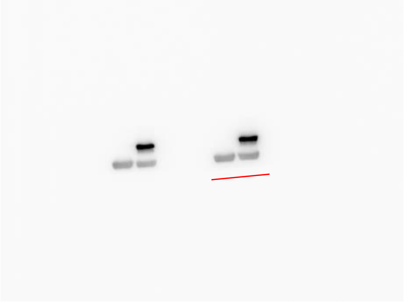

Figure 3F

Input ZNF582

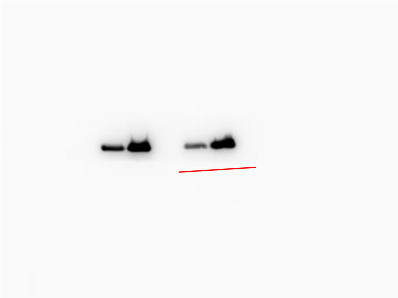

Input ZNF582-Flag

Input GAPDH

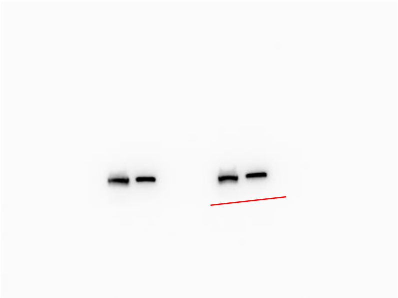

IP ERK2

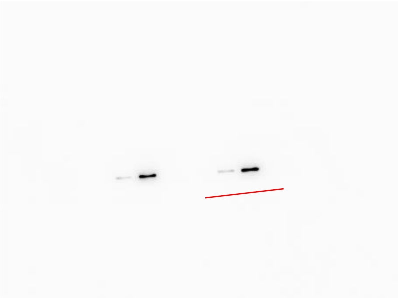

IP TJP2

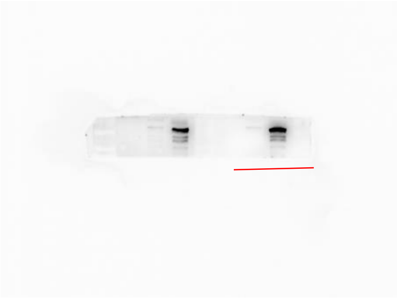

IP ZNF582

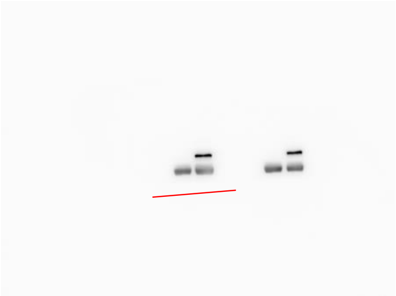

IP ZNF582-Flag

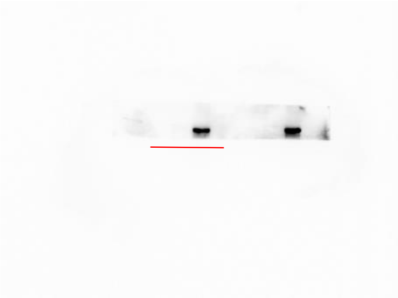

Figure 3G

ERK2

TJP2

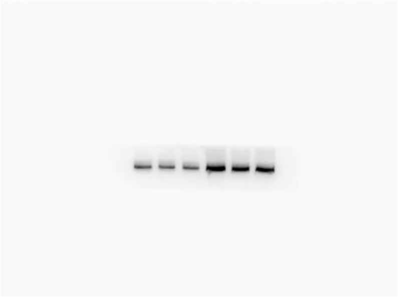

GAPDH

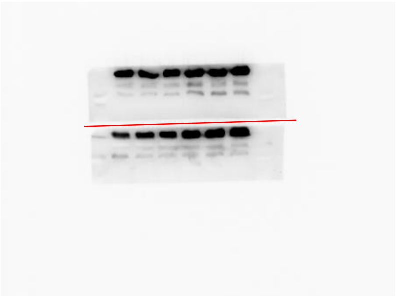

Figure 3H

ERK2

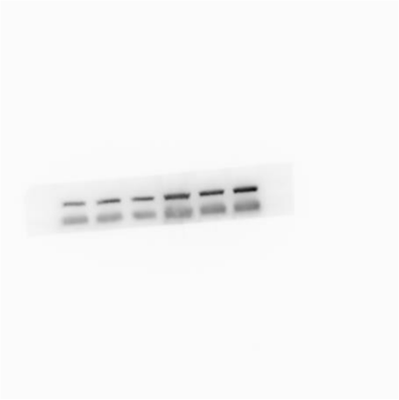

TJP2

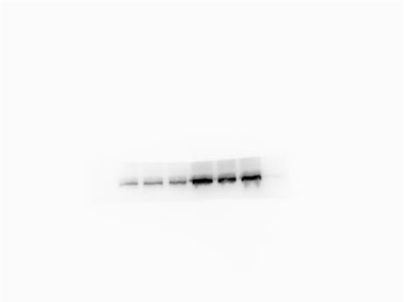

GAPDH

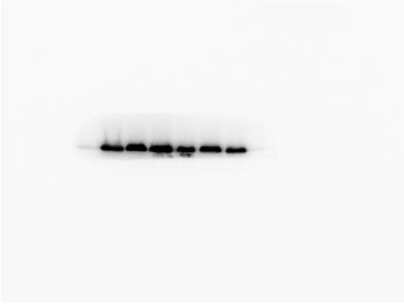

Figure 4A

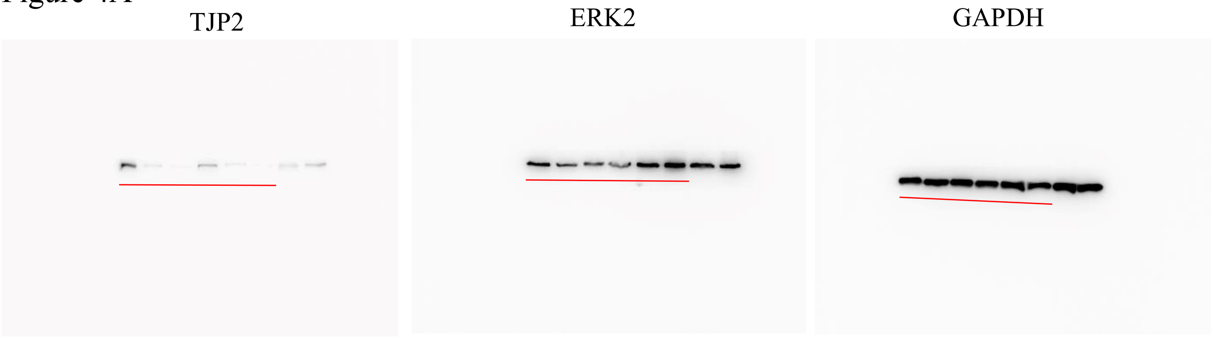

Figure 4B

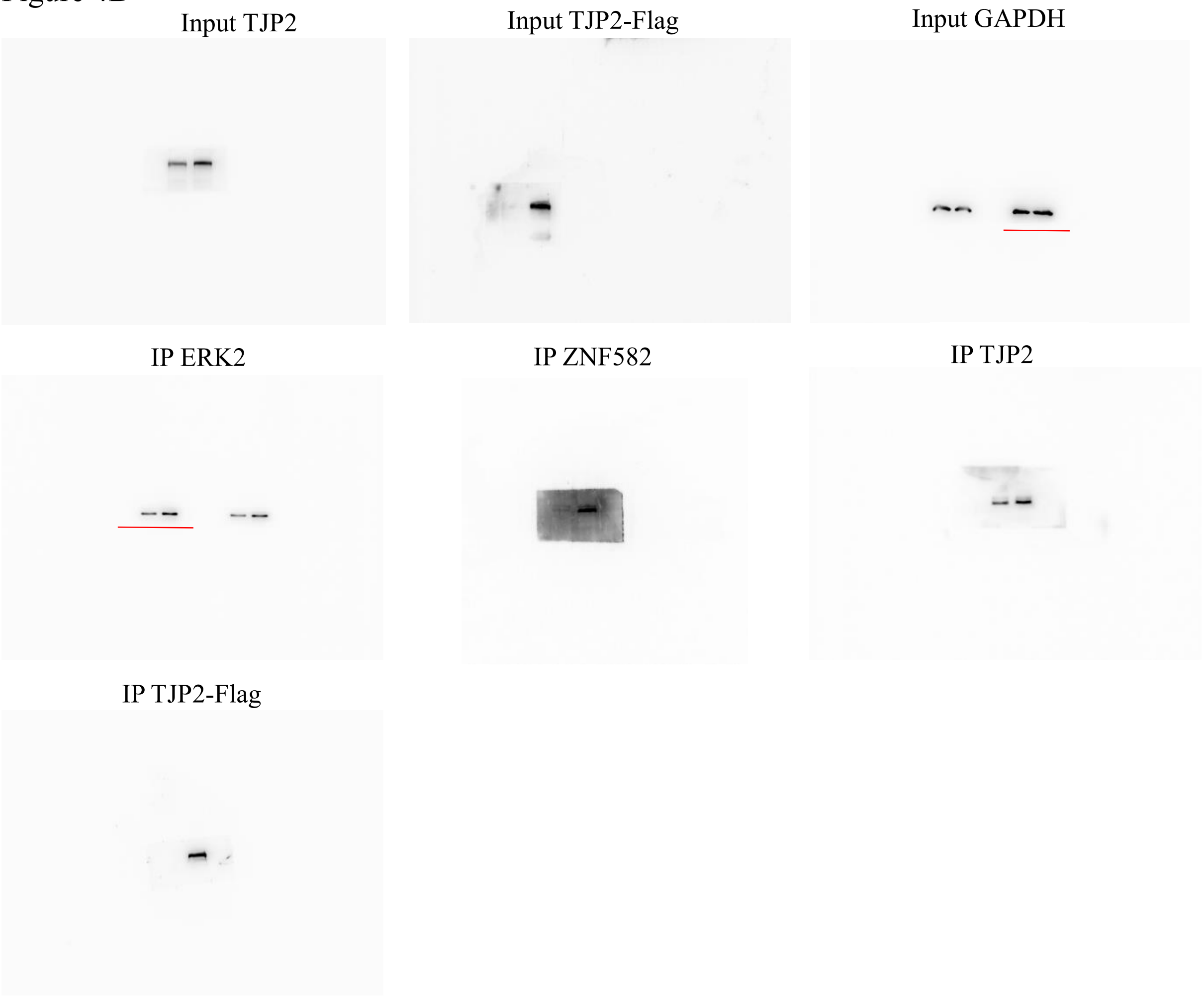

Figure 4C

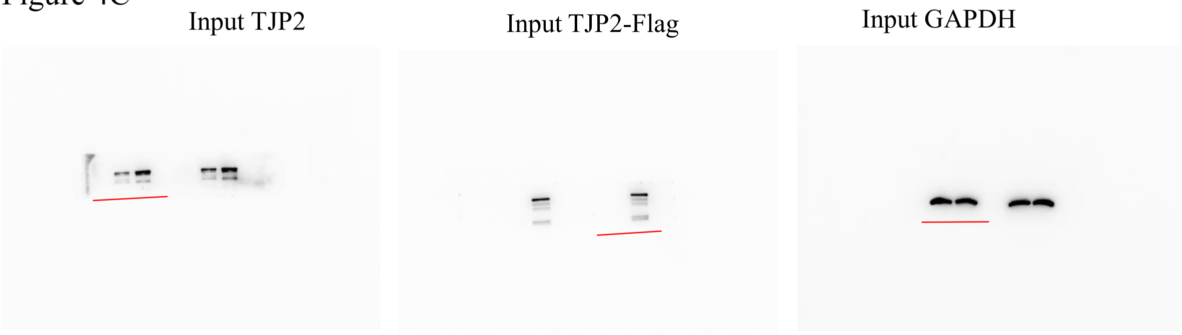

IP ERK2

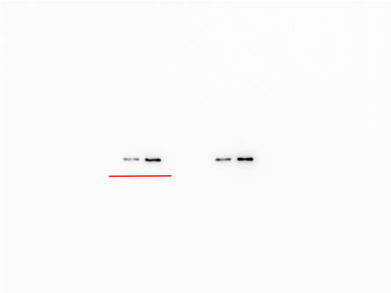

IP ZNF582

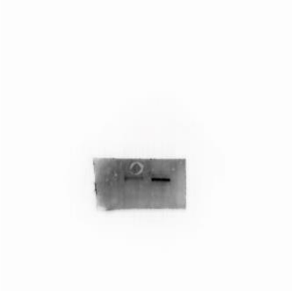

IP TJP2

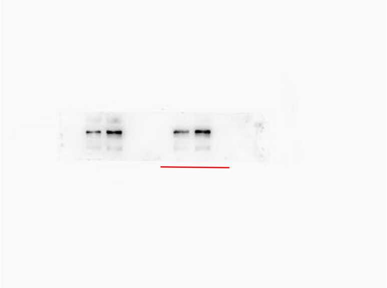

IP TJP2-Flag

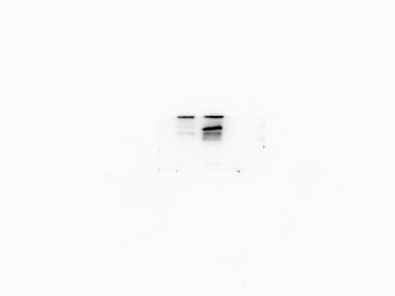

Figure 4D

TJP2

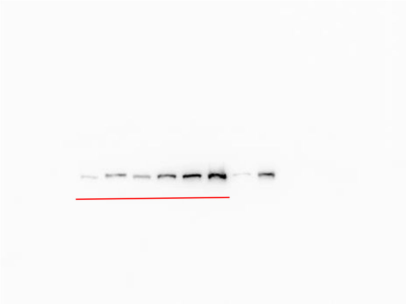

GAPDH

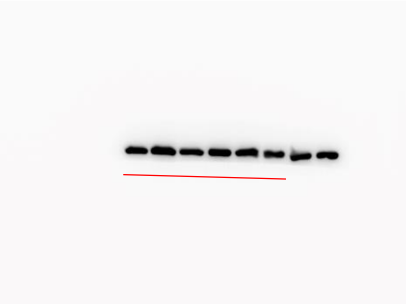

ERK2

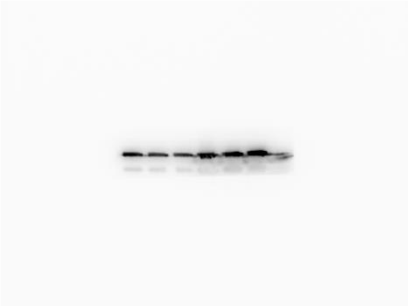

GAPDH

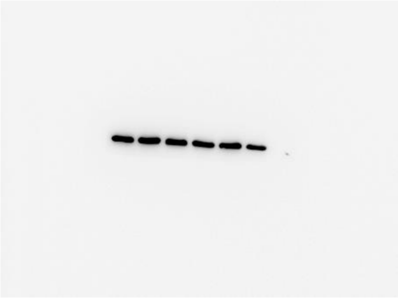

Figure 4E

TJP2

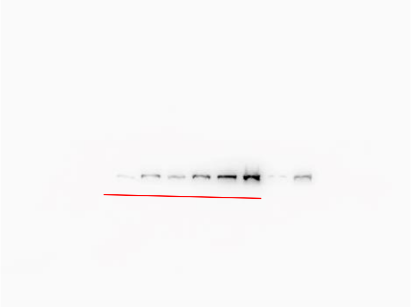

GAPDH

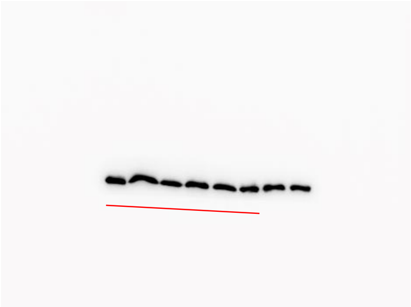

ERK2

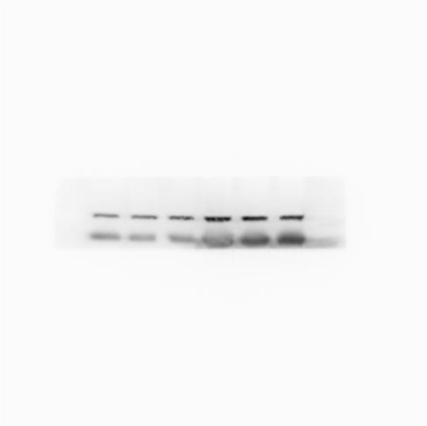

GAPDH

Figure 4F

TJP2

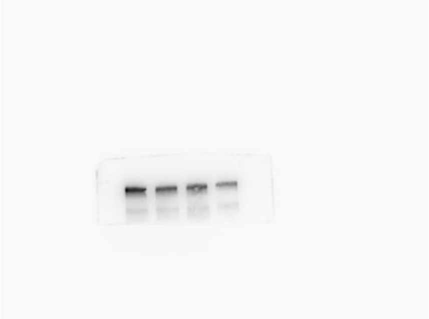

GAPDH

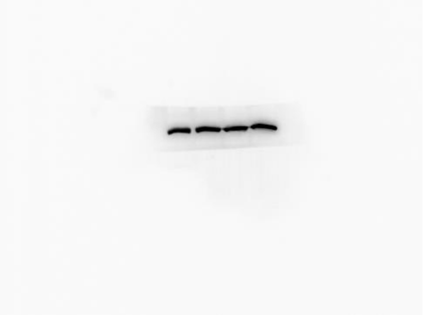

ERK2

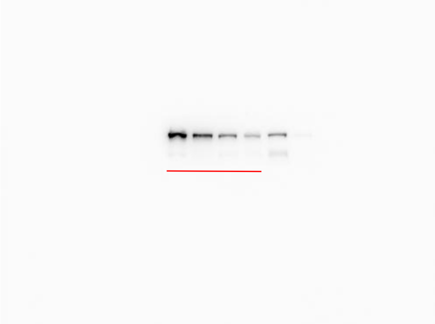

GAPDH

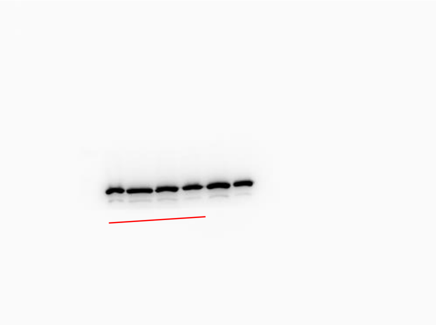

Figure 4G

TJP2

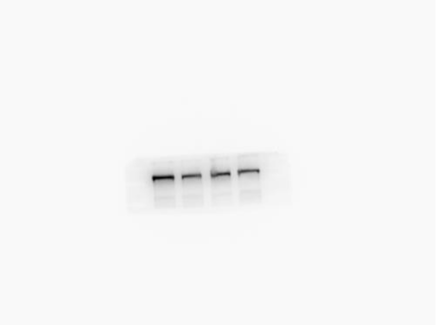

GAPDH

ERK2

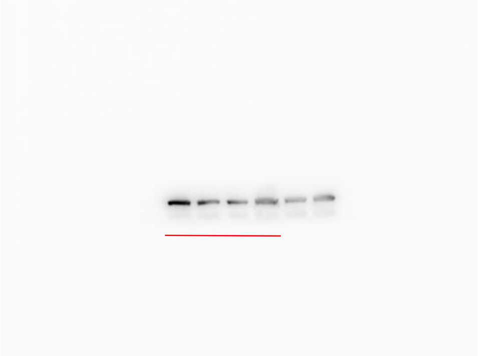

GAPDH

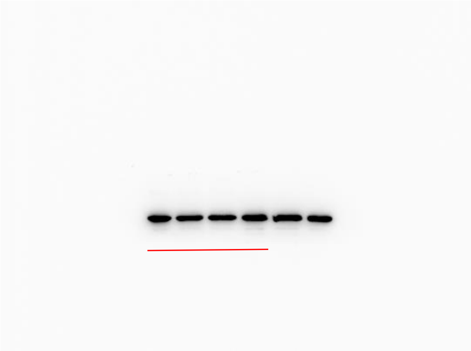

Figure 5A

p-ERK2

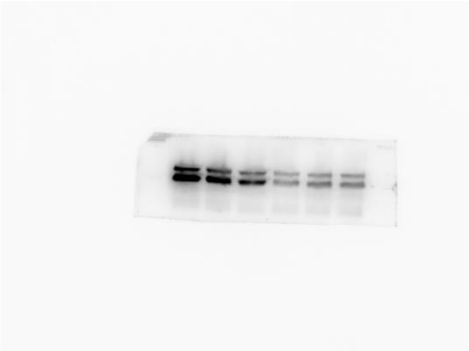

ERK2

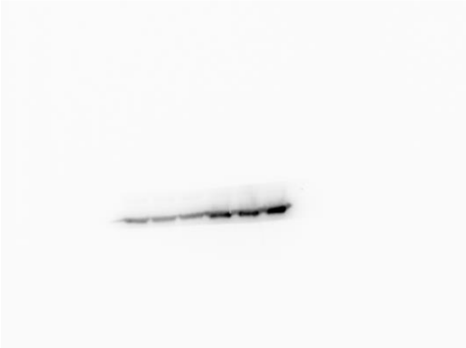

GAPDH

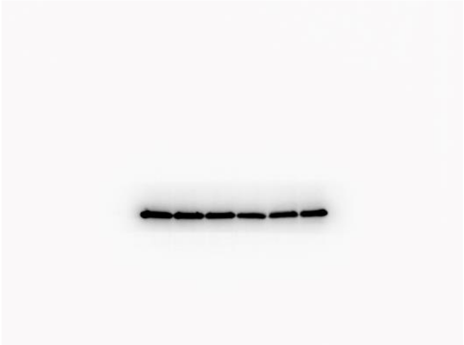

p-ERK2

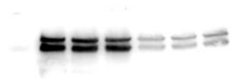

ERK2

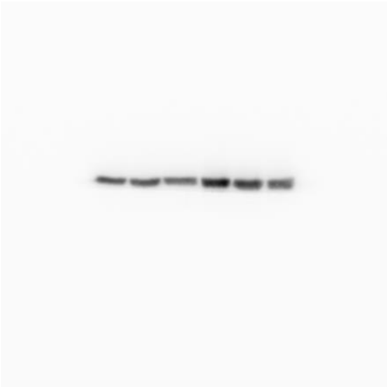

GAPDH

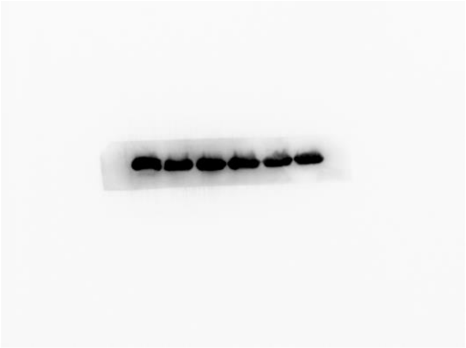

Figure 5B

p-ERK2

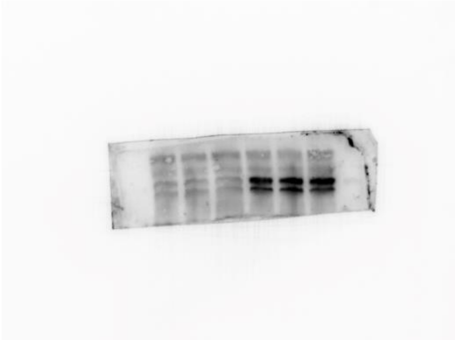

ERK2

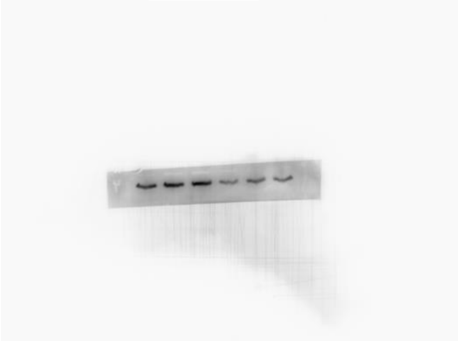

GAPDH

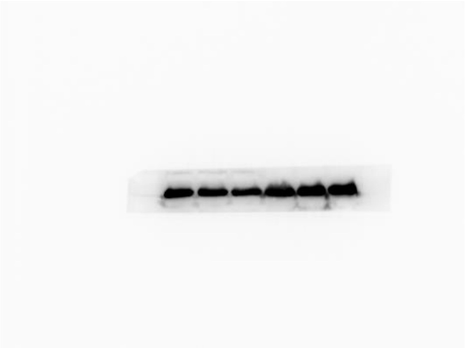

p-ERK2

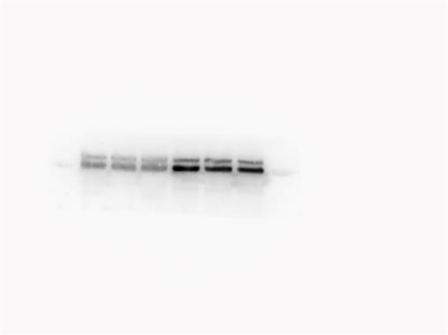

ERK2

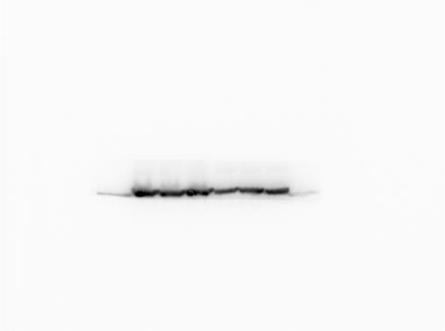

GAPDH

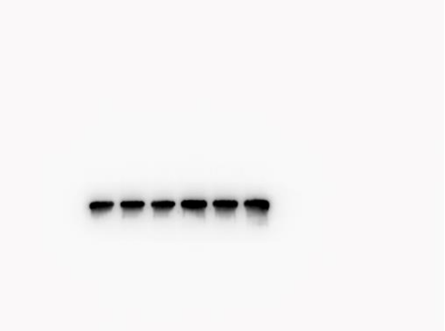

Figure 5C

MEK1/2

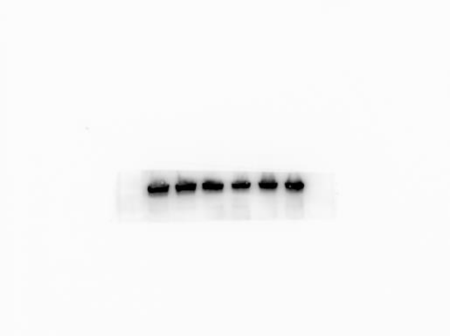

GAPDH

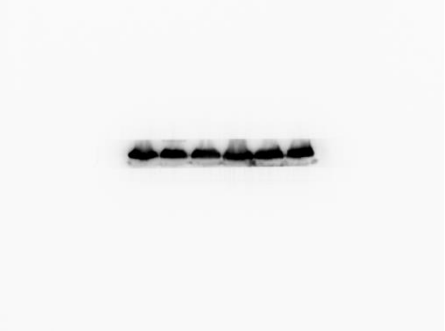

p-MEK1/2

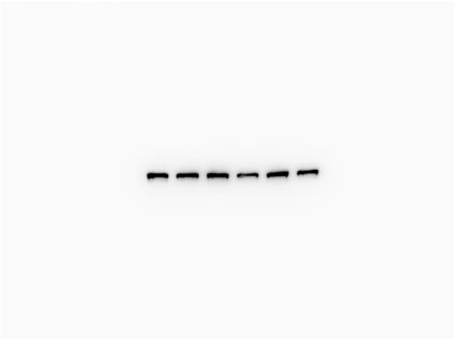

GAPDH

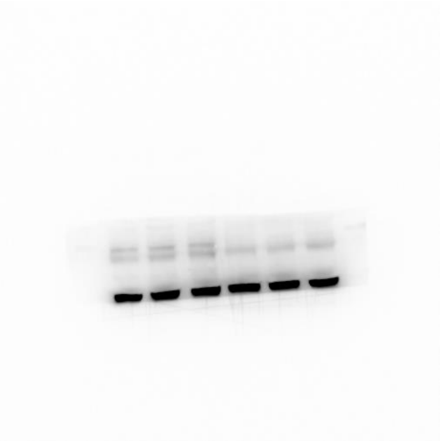

Figure 5D

MEK1/2

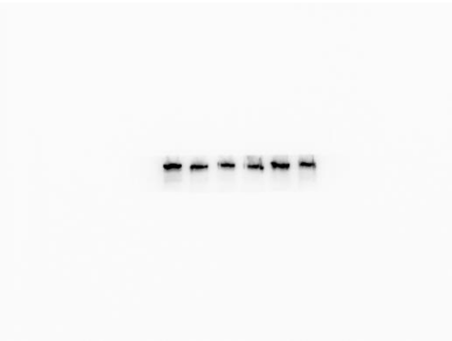

GAPDH

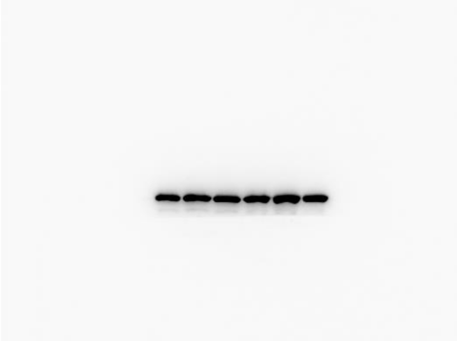

p-MEK1/2

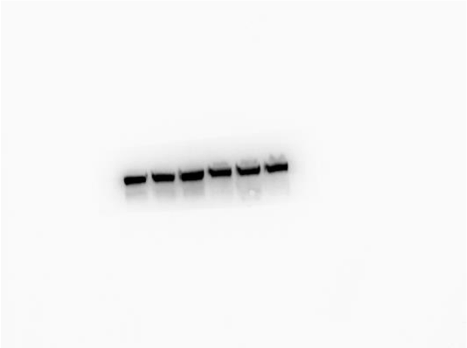

GAPDH

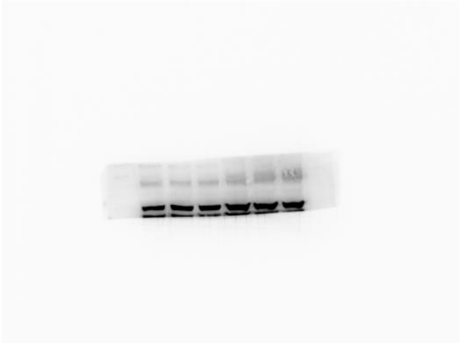

Figure 5E

Input

OSRC2
TJP2

Caki-1
TJP2

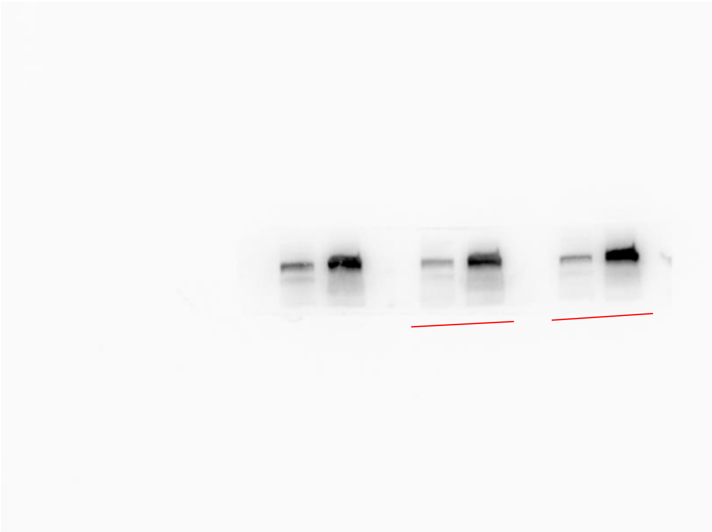

OSRC2
GAPDH

Caki-1
GAPDH

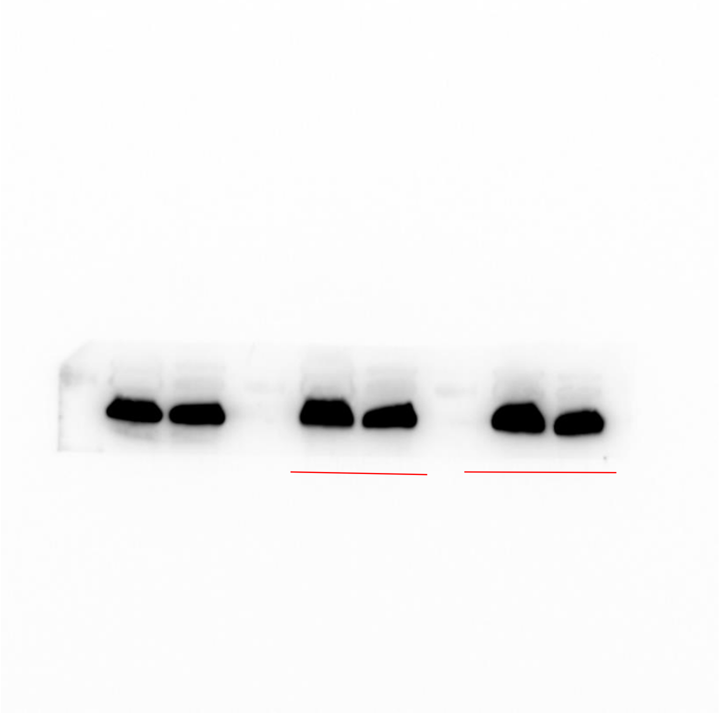

IP

OSRC2
ERK2

Caki-1
ERK2

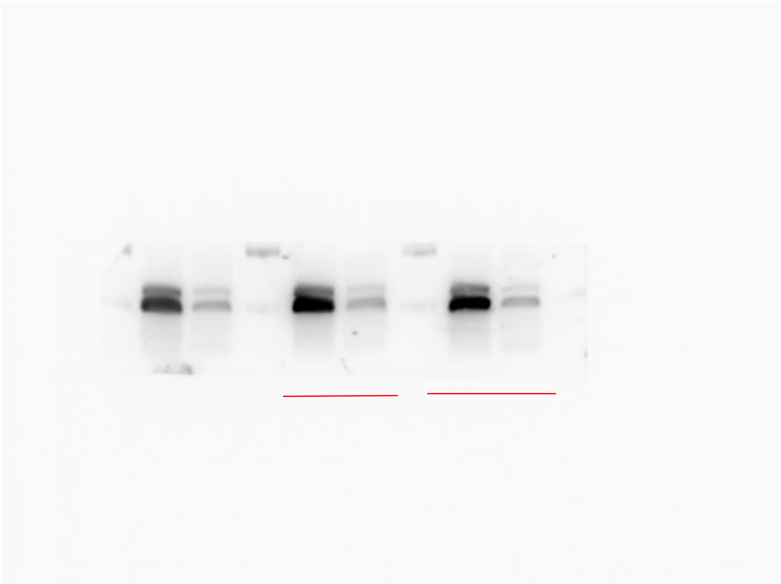

OSRC2 MEK1/2

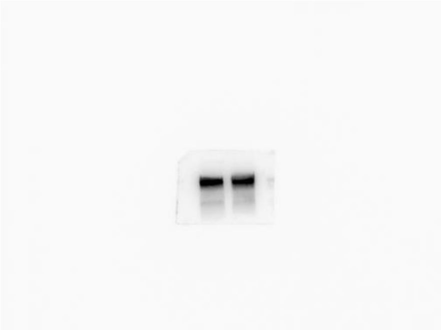

Caki-1 MEK1/2

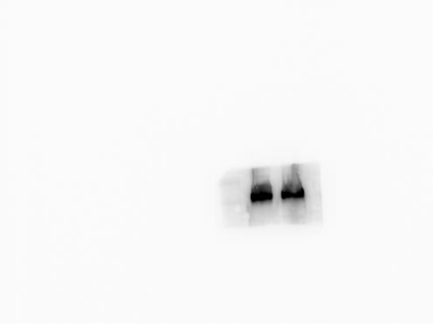

Figure 5F

BCL-2

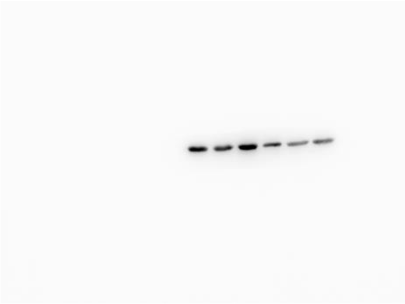

Cleaved Caspase-3

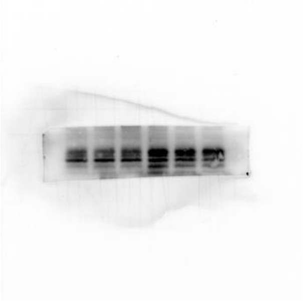

Caspase-3

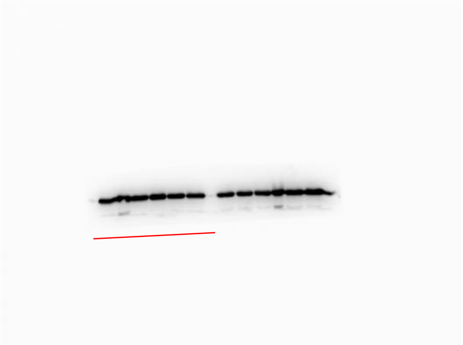

N-cadherin

E-cadherin

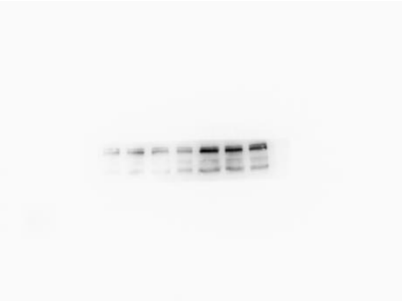

GAPDH

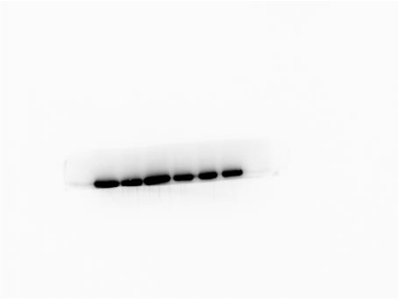

Figure 5G

BCL-2

Cleaved Caspase-3

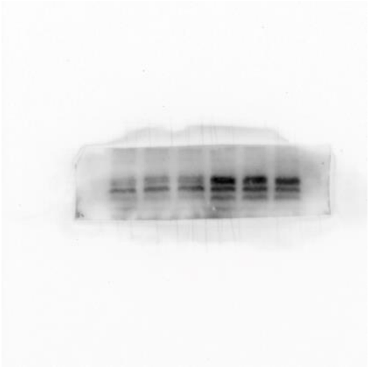

Caspase-3

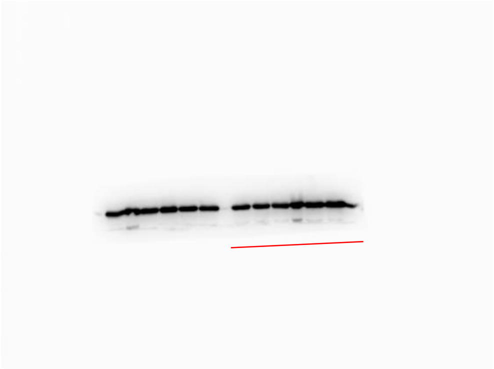

N-cadherin

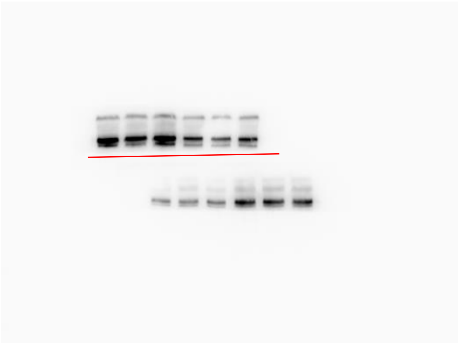

E-cadherin

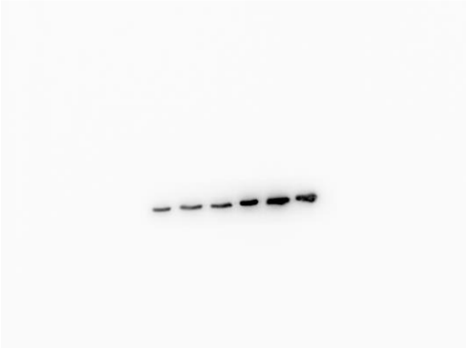

GAPDH

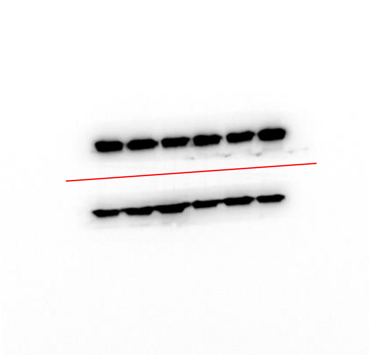

Figure 5H

BCL-2

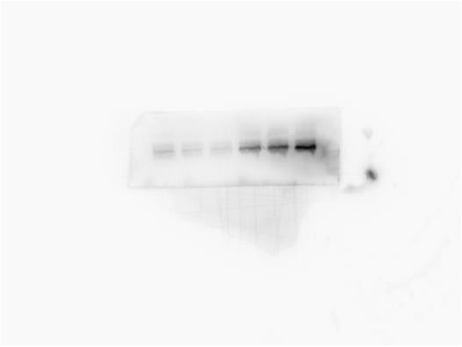

Cleaved Caspase-3

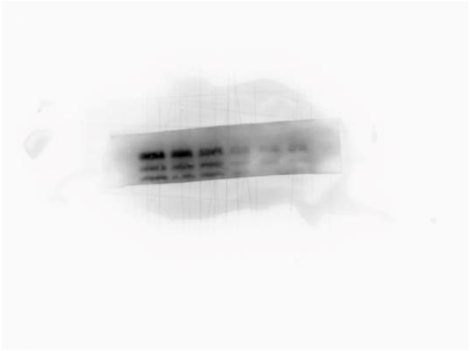

Caspase-3

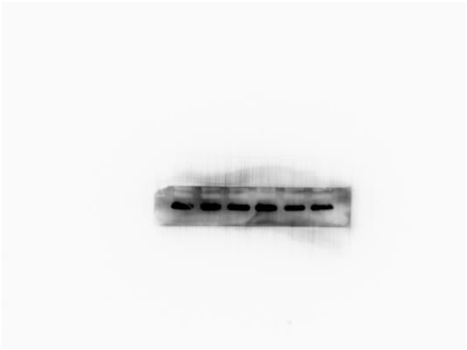

N-cadherin

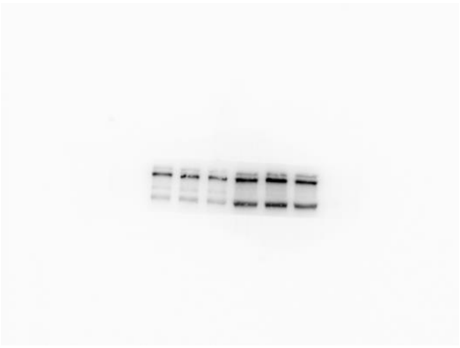

E-cadherin

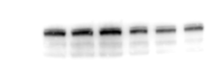

GAPDH

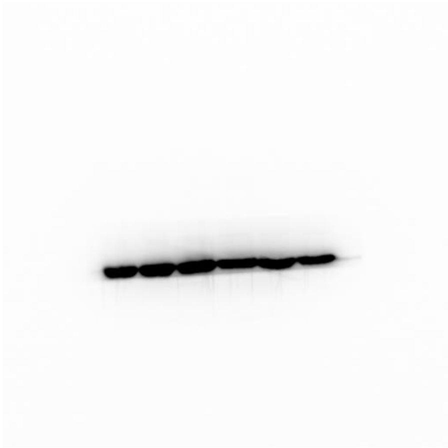

Figure 5I

BCL-2

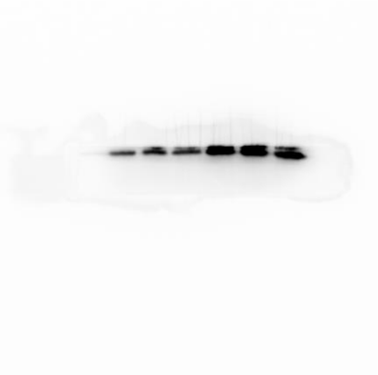

Cleaved Caspase-3

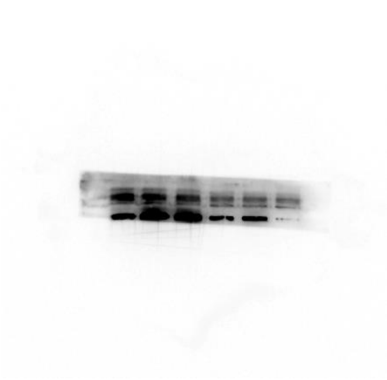

Caspase-3

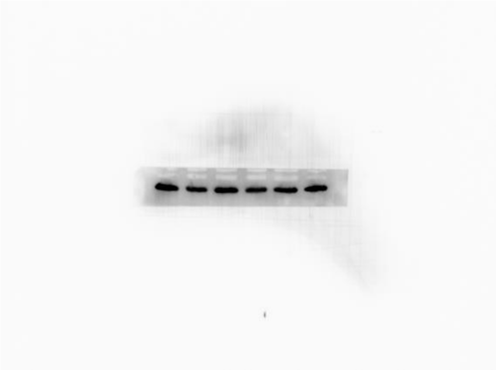

N-cadherin

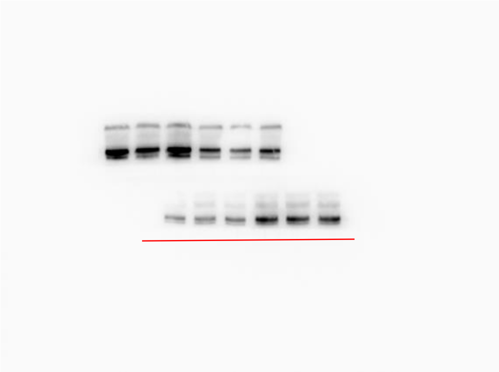

E-cadherin

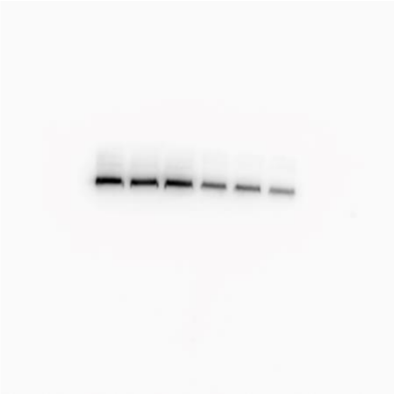

GAPDH

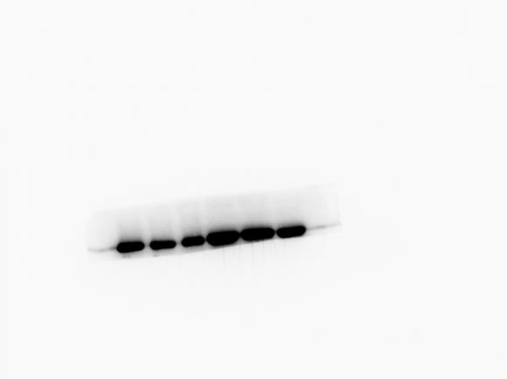

Figure 6H

TJP2 N1-T4

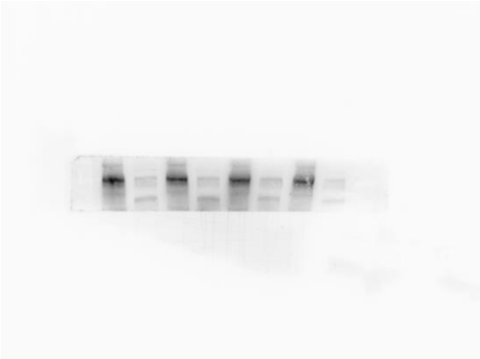

GAPDH N1-T4

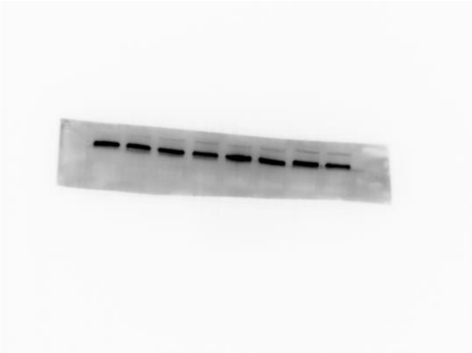

TJP2 N5-T8

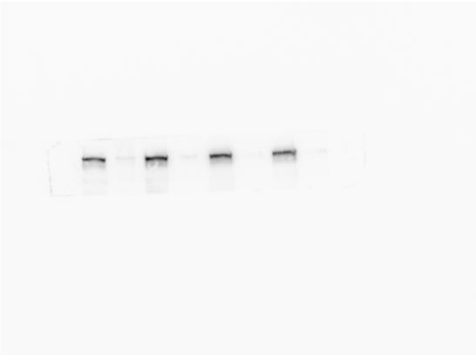

GAPDH N5-T8

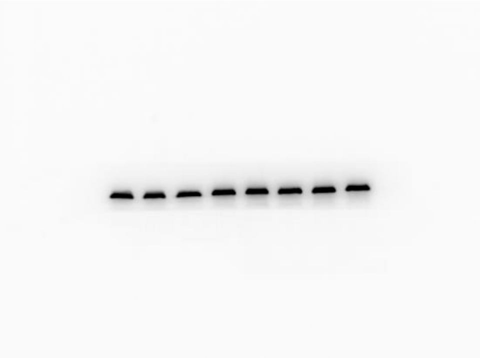

ERK2 N1-T4

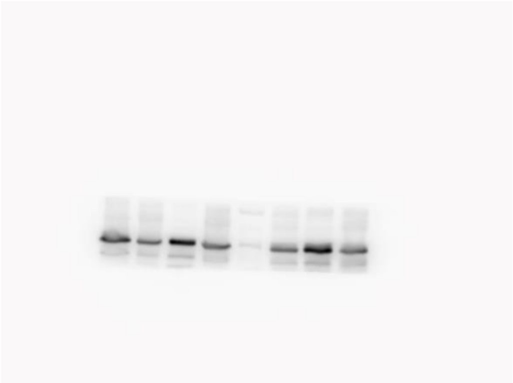

GAPDH N1-T4

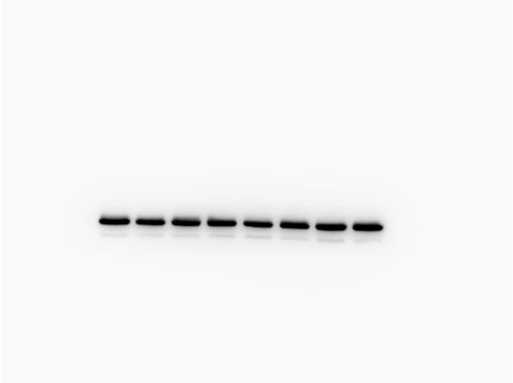

ERK2 N5-T8

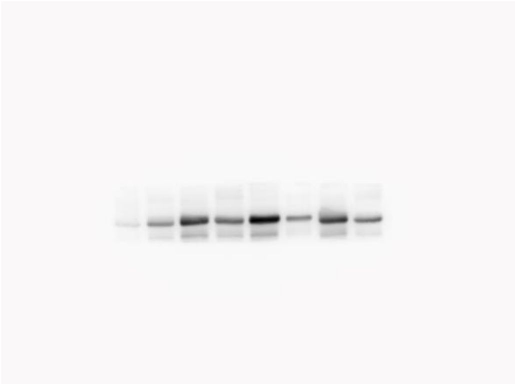

GAPDH N5-T8

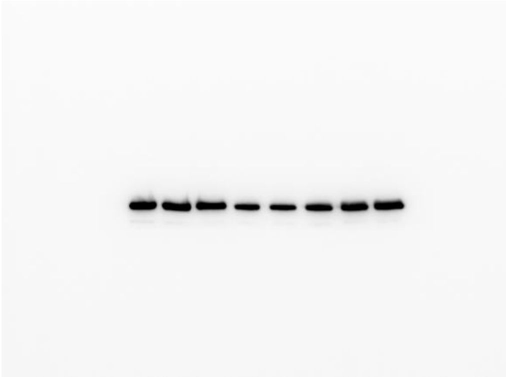

Supplemental Figure 5A

TJP2

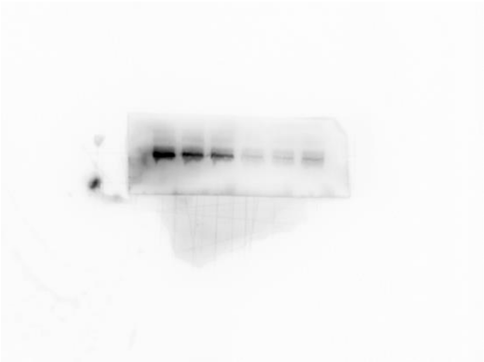

GAPDH

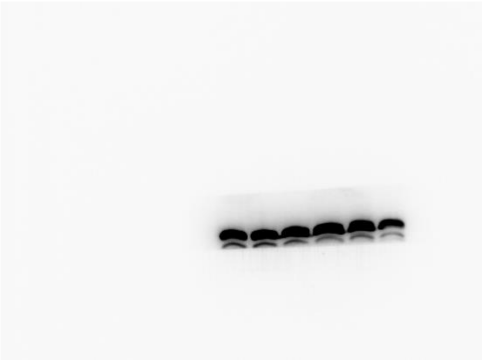

Supplemental Figure 5B

TJP2

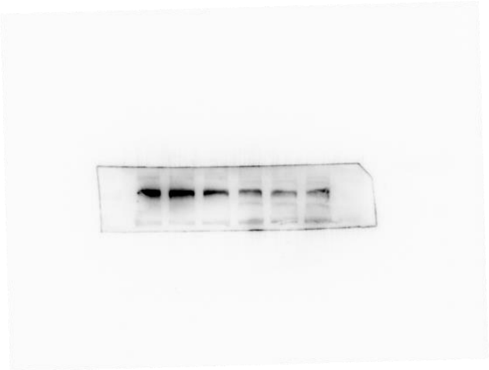

GAPDH

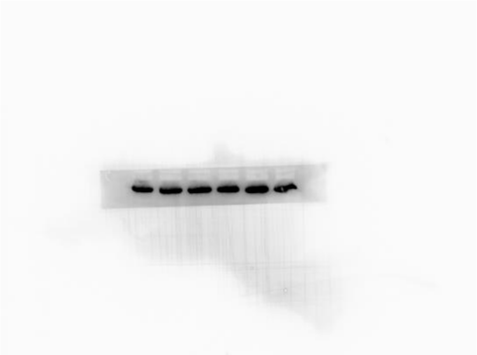

Supplement: Supplementary file 4 — Original Data File [file 41419_2023_5750_MOESM4_ESM.pdf]
